# Orthogonal and Multiresponsive Quinolinone Systems for Reversible and Recyclable Polymer Networks

**DOI:** 10.1021/jacs.5c08976

**Published:** 2025-08-27

**Authors:** Claas-Hendrik Stamp, Annalena Groß, Aitana Beato Irulegui, Bizan N. Balzer, Céline Calvino

**Affiliations:** † 685862 Albert Ludwig University of Freiburg, Cluster of Excellence livMatS, Georges-Köhler-Allee 105, Freiburg D-79110, Germany; ‡ Albert Ludwig University of Freiburg, Department of Microsystems Engineering (IMTEK), Georges-Köhler-Allee 102, Freiburg D-79110, Germany; § Albert Ludwig University of Freiburg, Institute of Physical Chemistry, Albertstr. 21, Freiburg D-79104, Germany; ∥ Albert Ludwig University of Freiburg – Freiburg Materials Research Center (FMF), Stefan-Meier-Str. 21, Freiburg D-79104, Germany

## Abstract

Achieving precise control over covalent bond formation and cleavage is critical for advancing material recycling and enabling repeated reuse. Here, we introduce quinolinones as versatile, multistimuli-responsive motifs enabling orthogonal and controlled covalent bond manipulation via a reversible [2π + 2π] cycloaddition triggered by light and thermal stimuli. While photochemical bond formation is well-established, thermal reversion of such bonds for material deconstruction remains underexplored. Furthermore, we demonstrate, for the first time, the exceptional ability of quinolinones to undergo symmetrical thermal cleavage, in the solid state with unprecedented efficiency, achieving over 99% monomer recovery at 210 °C within 10 min. This circular process is initially demonstrated at the molecular level, showing an effective cyclability of at least three full bond formation and cleavage cycles with quantitative efficiency. Extending to the macromolecular scale, quinolinones are incorporated into linear polymers to enable phototriggered network formation followed by thermally induced bulk deconstruction. This responsive polymer system highlights remarkable versatility and recyclability. Finally, exploiting their multiresponsivity, quinolinones are applied to advanced coatings with reversible debonding and thermal degradation capabilities. This work establishes quinolinones as a robust platform for stimuli-responsive materials, paving the way for next-generation recyclable systems with enhanced functionalities.

## Graphical abstract

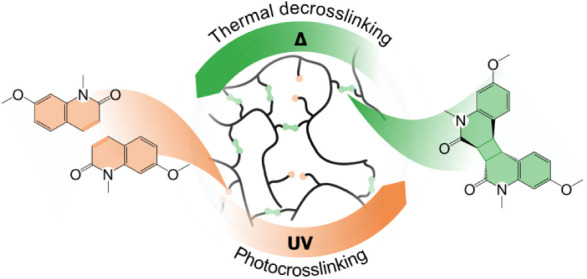

## Introduction

The growing demand for sustainable materials has catalyzed innovation in designing soft materials capable of controlled deconstruction or de-cross-linking for efficient reprocessing.^1–5^ In recent years, covalent adaptive networks—polymer systems incorporating dynamic covalent bonds that reversibly form and break in response to stimuli such as heat—have emerged as a promising strategy for bulk material recovery.^6–8^ At elevated temperatures, these systems undergo reversible bond exchange, driving macromolecular transitions such as flow and stress relaxation, key features that allow reshaping and reuse.^9–11^ Recently, attention has shifted toward achieving greater control over these dynamic bonds to enable independently triggered and permanent structural transformations. Such systems would allow materials to be assembled and disassembled on demand, making recycling more effective and compatible with various processing environments.^12,13^

Despite advances in the field, only a limited number of reactions offer truly controllable and reversible bond formation and cleavage under external stimuli.^13,14^ Among these, photoswitchable [2π + 2π] pericyclic reactions stand out for their spatial and temporal precision, enabling reversible covalent transformations with high selectivity.^15–17^ These systems typically operate through the light-induced cycloaddition of conjugated alkenes to form cyclobutane adducts, which can subsequently be cleaved by irradiation at a different wavelength (Figure 1a).^15,18^ This reversible mechanism has been harnessed to mediate reversible crosslinking in a range of polymeric materials, enabling light-triggered gelation,^19^ surface healing,^20^ DNA binding,^21^ photoactuation,^22^ and functional coatings,^23^ among other applications. Despite offering precise and reversible bond formation, the use of [2π + 2π] photocycloaddition in reversible polymerizations and bulk reprocessing still remains limited, primarily due to inefficiencies in the cleavage (reversion) step. Under continuous irradiation, these systems quickly reach a photostationary state where forward and reverse processes compete due to spectral overlap between the cyclobutane product and the original alkene monomers.^12,24^ As a result, photocleavage yields in bulk materials are typically below 40%,^15,24–27^ preventing complete material deconstruction. Compounded by poor overall conversions (<80%) and limited light penetration in thick or opaque samples, these constraints pose serious barriers to practical implementation.^28,29^ To overcome these limitations, several groups have explored thermal cleavage of [2π + 2π] adducts, traditionally considered “forbidden” under Woodward–Hoffmann rules.^30,31^ Early studies revealed that simple cyclobutanes, such as those formed from ethylene, undergo thermal dissociation through a well-established stepwise biradical mechanism.^32–34^ This foundational work has since inspired efforts to investigate thermally induced cleavage of cyclobutane units embedded in more complex chromophores. At the molecular level, these reactions offer a thermally activated pathway that complements photocycloaddition, enabling reversible dimer–monomer cycling in responsive systems.^35–37^ Yet, despite promising molecular examples, thermal reversion for polymer matrix deconstruction remains largely unexplored. A notable advancement was reported by Houck and coworkers, who introduced a thiomaleimide moiety capable of orthogonal, light-activated [2π + 2π] dimerization under 350 nm irradiation, followed by thermally induced cleavage above 120 °C.^38^ This system demonstrated exceptional versatility, allowing quantitative bonding and debonding cycles in both solution and bulk. When incorporated in multifunctional building blocks, the photoprocess converted viscous liquids into crosslinked networks that could be thermally depolymerized back to their original state. While this work highlights the feasibility of coupling light-induced assembly with thermal deconstruction, it remains among the very few examples demonstrating reversible [2π + 2π] dimerization in bulk polymer matrices with clear structural characterization. It is worth noting that another well-known example of reversible photodimerization and thermal cleavage in polymer systems involves [4π + 4π] cycloaddition reactions, such as those of anthracene.^39^ Anthracene-based materials demonstrate combined photo- and thermoreversibility, with photodimerization typically induced under UV light (∼350 nm) and thermal cleavage occurring around 100–150 °C.^40^ These conditions provide a useful benchmark for dynamic covalent crosslinking in polymers, offering processing windows compatible with many thermoplastics. However, while anthracene dimers are highly stable, they often show limited reactivity in the solid state and incomplete reversibility in bulk materials. These limitations emphasize the ongoing need for alternative photothermally reversible chemistries with improved stability and more tunable processing conditions.

**Figure 1:**
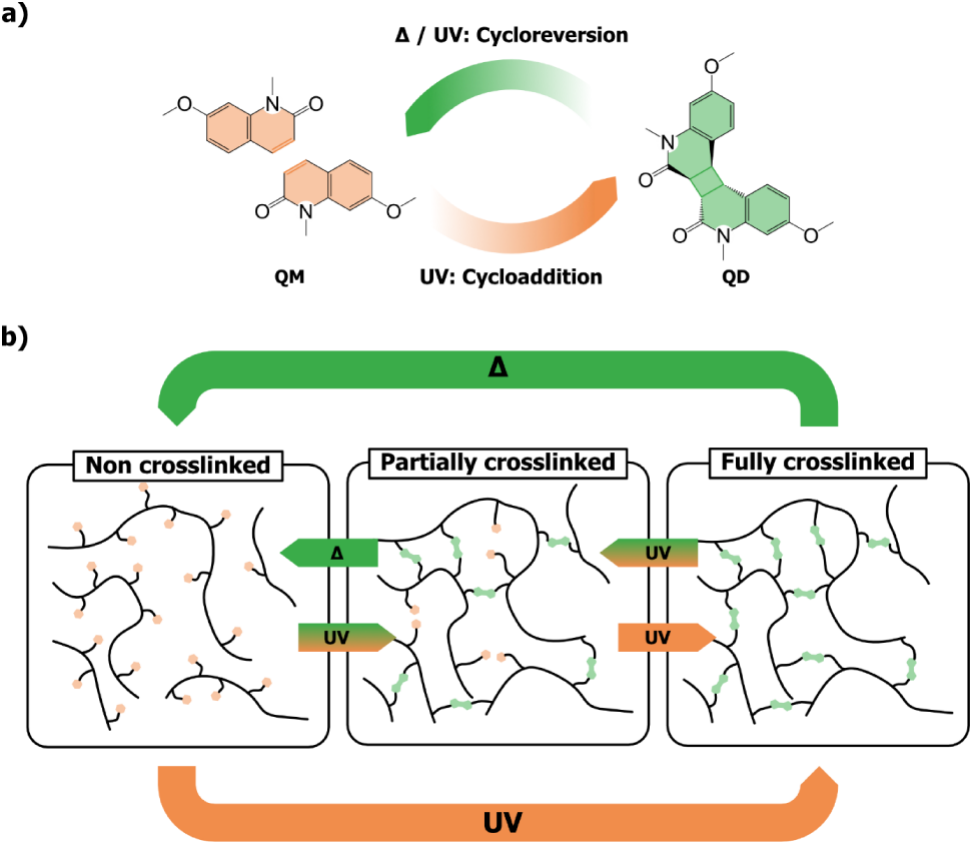
a) Schematic representation of UV-driven [2π + 2π]-photocycloaddition and thermally (Δ) or UV-driven symmetric reversion of quinolinone. b) Illustration of multiresponsive polymer network based on quinolinone moieties.

Motivated by the scarcity of alternative systems, we turned to a class of chromophores previously developed in our group: quinolinone-based motifs.^41^ These compounds share structural similarities with coumarins, which have been extensively studied for reversible [2π + 2π] photoreactions in soft materials.^42–44^ Despite some reports of thermal cleavage at the molecular level, to the best of our knowledge, no clear evidence exists for clean, symmetric thermal bond scission of coumarin dimers in solid-state polymer networks, which typically exhibit high thermal stability up to 300 °C depending on their configuration.^42^ As an alternative, quinolinones present a compelling scaffold: by replacing the coumarin lactone with a lactam, they introduce electronic and conformational changes that both enhance photoreactivity and destabilize the resulting dimers.^45–49^ These features led us to hypothesize that quinolinone dimers might also exhibit reduced thermal stability, offering a route to thermal cleavage under practical conditions.

Here, we report the first example of clean, symmetrical and quantitative thermal cleavage of quinolinone dimers into their alkene monomers in the solid state. The recovered monomers retain full functionality and undergo multiple cycles of photochemical dimerization and thermal cleavage, demonstrating exceptional reversibility and responsiveness (Figure 1a).

On the macromolecular scale, this chemistry enables independent photochemical polymer network formation, followed by their thermal deconstruction in bulk (Figure 1b). Furthermore, the extent of photo-crosslinking and thermally induced decrosslinking can be precisely controlled by modulating irradiation wavelength, exposure time, and tempering duration. The solid-state performance of this orthogonal system highlights the exceptional versatility of quinolinones, enabling precise tuning of material properties and enhanced control, highlighting their strong potential for advanced functional material design.

## Results and Discussion

### Thermal Reversion at the Molecular Scale

As an initial step, the thermal properties of 7-methoxy-1-methylquinolin-2­(1H)-one (**QM**) and its quinolinone dimer (**QD**) (Figure 1a) were investigated at the molecular level in the solid-state. It is worth noting that, while previous work has shown that the photodimerization of quinolinones can, in principle, yield multiple regio- and stereoisomers, controlled irradiation conditions have been demonstrated to selectively produce the anti head-to-head isomer.^41^ This isomer, referred to as **QD**, was therefore exclusively obtained and used throughout the present study. To establish temperature ranges suitable for practical applications, the thermal stability of **QM** and **QD** was preliminarily assessed using thermogravimetric analysis (TGA). The resulting TGA curve of **QM** revealed a pronounced mass loss starting at 206 °C (Figure S1a). Given the possibility that **QM** may sublimate before undergoing thermal degradation, the compound was held at 206 °C for 2 h to assess whether mass loss was due to evaporation or chemical decomposition (Figure S1b), and the residue was subsequently analyzed by ^1^H NMR spectroscopy. A gradual mass loss of approximately 68% was observed in the TGA trace, while the NMR spectra confirmed the presence of intact **QM** and the absence of degradation products (Figure S2). These results indicate that the observed mass loss is primarily due to sublimation rather than thermal decomposition, confirming the thermal stability of **QM** under these conditions. Building on this, **QM** was subjected to multiple heating and cooling cycles between 25 and 170 °C at a rate of 5 °C/min using a differential scanning calorimeter (DSC) (Figure 2a). In the first two cycles, the thermal profile revealed an endothermic transition at 101 °C, likely corresponding to the melting of **QM**, followed by an exothermic transition at 83 °C during the cooling ramp, consistent with recrystallization. The absence of additional transitions and the consistent integrated values of the transition enthalpy confirmed that **QM** remains stable and does not degrade during these thermal processes. Interestingly, when heated above 200 °C a downward shift in the crystallization temperature (from 77 to 70 °C) was observed across subsequent DSC cycles, while integrated enthalpy values remained unchanged (Figure S3a-b), and the melting transition remained stable. This behavior is attributed to kinetic effects, where under small-scale DSC conditions, discrete droplets may form and undergo varying degrees of supercooling. This behavior results in shifts in crystallization temperature and multiple recrystallization events.^50–52^ These variations do not indicate degradation but instead reflect reproducible morphological changes and nucleation-driven phenomena.

**Figure 2:**
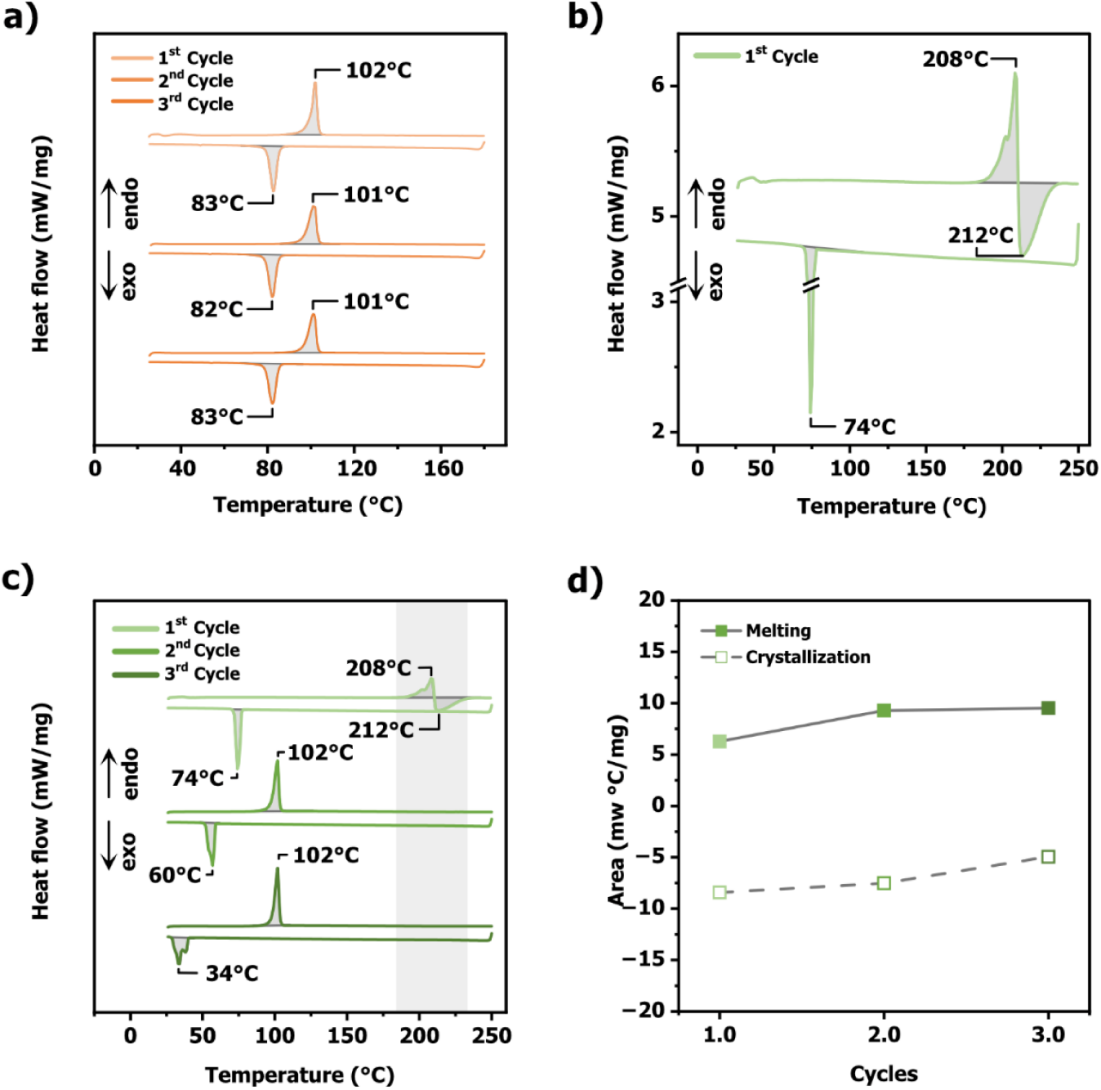
a) Differential scanning calorimetry (DSC) traces showing three consecutive heating and cooling cycles of **QM** recorded from 25 to 170 °C at a rate of 5 °C/min. b) DSC traces of the first heating and cooling cycle of **QD** recorded from 25 to 250 °C at a rate of 10 °C/min. c) DSC traces depicting three consecutive heating and cooling cycles of **QD**. d) Comparison of the melting and crystallization peak areas as a function of the DSC cycle shown in c).

Similar to **QM**, the thermal profile of **QD** was assessed through TGA, revealing thermal stability up to 199 °C (5% weight loss) (Figure S4). Complementary DSC measurements conducted between 25 and 300 °C identified an endothermic transition at 208 °C, likely corresponding to dimer melting, followed by a broad exothermic transition at 212 °C (Figure 2b). Additionally, during the cooling cycle, a crystalline transition was detected at 74 °C. Notably, in the following heating cycle (Figure 2c), a new melting transition at 102 °C, consistent with the monomer’s melting point, was observed, while the endothermic and exothermic events previously seen at 208 and 213 °C were no longer present. Furthermore, as seen in the DSC traces of **QM**, similar downward shifts in crystallization temperatures were noted, while the melting temperature and the area under all transitions remained constant (Figure 2c,d).

The observed thermal sequences, particularly the exothermic transition, strongly suggest structural changes within the molecule, most likely associated with the cleavage of the cyclobutane bond, as previously reported in related studies.^53^ This interpretation is reinforced by the recovery of thermal signatures closely matching those of the parent monomer **QM** and further confirmed by ^1^H NMR analyses conducted after the third heating and cooling cycle (Figure S5a,b). Specifically, the disappearance of cyclobutane proton signals at 3.71 ppm, the appearance of new alkene signals at 7.62–7.58 ppm and 6.57–6.54 ppm, and the overall shift in remaining resonances indicate the regeneration of **QM** (Figure 3a,b). Collectively, this robust evidence confirmed a clean, quantitative, and symmetrical reversion of the cyclobutane unit to the original monomer **QM**, with no detectable side products, showcasing the precision and efficiency of this transformation.

**Figure 3:**
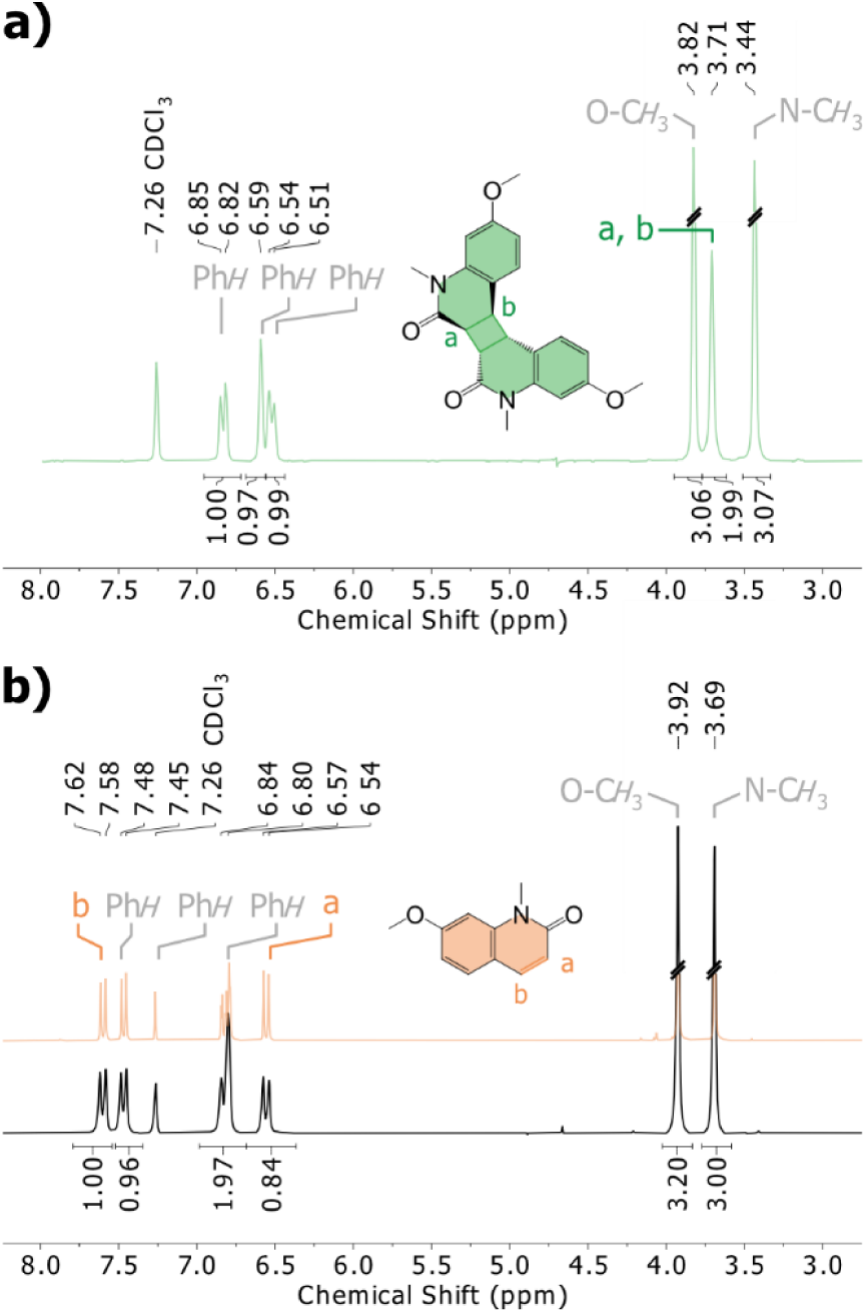
a) ^1^H NMR spectrum (250 MHz, 297.2 K, CDCl_3_) of quinolinone dimer (**QD**). b) ^1^H NMR spectrum of **QD** (black spectra) measured after the first heating and cooling DSC cycle (temperature range: 25–250 °C, heating/cooling rate: 10 °C/min). ^1^H NMR spectrum of **QM** (orange) is illustrated as comparative reference.

To the best of our knowledge, this system represents the first report of symmetrical and fully reversible thermal cleavage of cyclobutene-linked quinolinones to their parent monomers. This thermally reversible process, combined with the previously demonstrated orthogonal photoreversible behavior,^41^ establishes quinolinone dimers as a rare example of a multiresponsive system capable of precise, stimuli-controlled bond manipulation.

Following confirmation of the thermal reversibility of **QD**, kinetic studies were conducted to determine the activation energy and reaction rates associated with the thermal cleavage process. Establishing these parameters is essential for predicting and controlling the conditions under which the transformation occurs, thereby enabling precise thermal management for potential applications. To this end, solid-state samples of **QD** were heated at incremental temperatures of 180 °C, 190 °C, 200 °C, and 210 °C and analyzed by^1^H NMR at time intervals ranging from 30 s to 30 min (see details in the Supporting Information, Figure 4a). Conversions determined from integration of the corresponding spectra exhibited first-order kinetics across all measured temperatures (Figures S6–S10a), with half-life times ranging from 1 to 37 min (Figure 4b). The activation energy for the thermally induced bond cleavage was calculated to be 200 (±22) kJ/mol, from the slope of the Arrhenius plot (ln­(k) vs (1/T)), based on the experimentally determined rate constants (Figure S10a-c). Notably, this value aligns closely with previously reported activation energies for thermal reversion of dimers formed through the photocycloaddition of photoswitchable motifs, such as thiomaleimide (134 kJ/mol)^39^ and anthracene (155 kJ/mol).^54^ These results pinpoint the effective temperature range for triggering the thermal bond scission cloreversion of **QD**, starting at 180 °C. As the temperature increases, the time required for full conversion decreases substantially—from approximately 2 h at the lower threshold to just 10 min at higher temperatures (Figure 4b). This temperature-dependent tunability highlights both the efficiency and controllability of the **QD** thermal cleavage process, demonstrating its suitability for applications requiring precise thermal activation. Importantly, the thermally reverted **QD** was successfully reused for photodimerization in solution under UV irradiation at 340 nm (*c* = 6.5 × 10^–5^ mol/L), exhibiting reactivity comparable to that of the pristine quinolinone monomer (**QM**), which had previously demonstrated efficient dimerization under analogous conditions (Figure S11a–b).^41^

**Figure 4:**
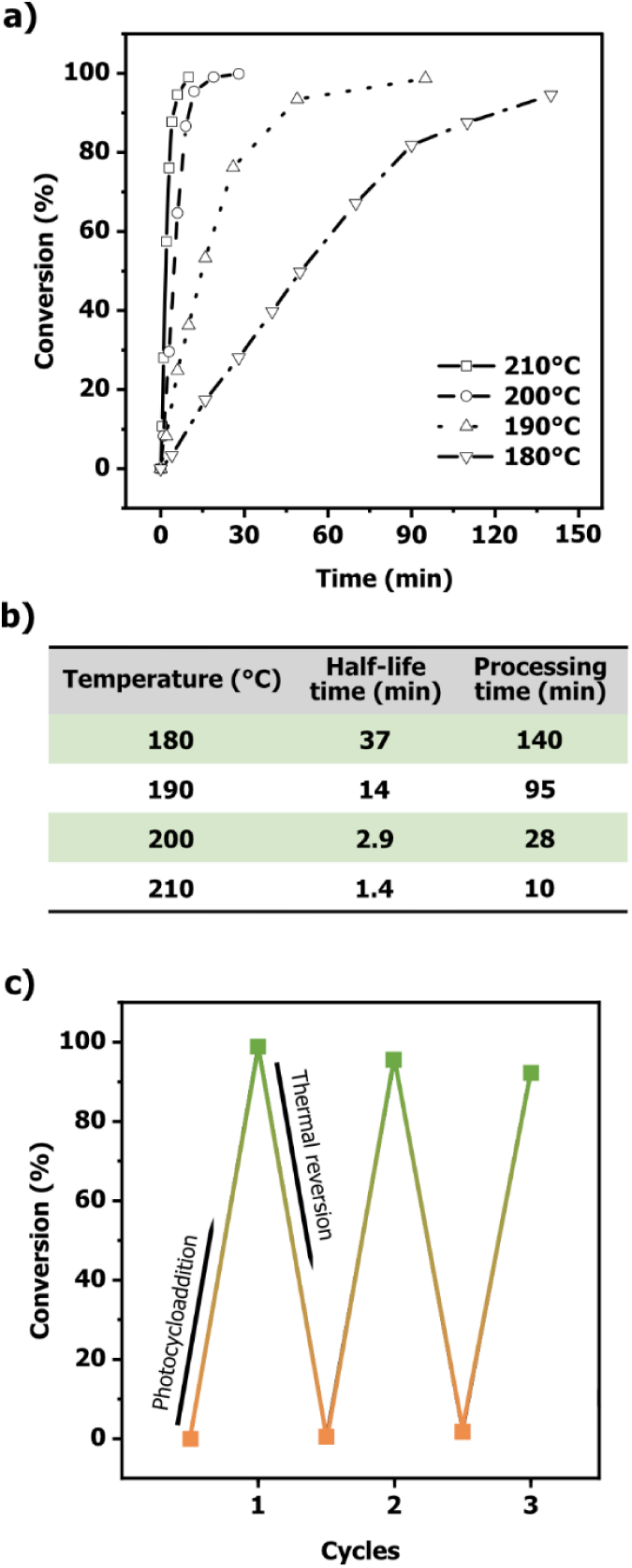
a) Thermal cleavage conversions of **QD** in the solid state under oxygen-free conditions at temperatures of 180, 190, 200, and 210 °C. b) Calculated half-life values for thermal cleavage at each temperature, and processing times required for complete reversion of **QD** at the respective temperatures. c) Reaction conversion over three photothermal processing cycles. Photocycloaddition reactions were conducted in solution at *c* = 6.5 × 10^–5^ mol/L, followed by thermal reversion in the solid state.

These results confirm that the quinolinone motif fully retains its photochemical reactivity after thermal reversion, underscoring its exceptional chemical robustness. The system was subsequently subjected to three complete cycles of photodimerization in solution followed by thermal cleavage in the solid state and showed only a slight decrease in the conversion back to the dimer, with no apparent loss in reactivity (Figure 4c) presumably due to manipulation-related losses such as partial evaporation of the monomer during heating. This reproducible cycling behavior highlights the reliability of the system and establishes quinolinones as a versatile, orthogonally addressable molecular platform for the design of recyclable and thermally responsive photopolymers.

To assess the applicability of the system in bulk materials, both the photo- and thermally induced transformations were tested in solid-state molecular blends. Accordingly, **QM** was incorporated at 1 wt % into a commercially available poly­(methyl methacrylate) (PMMA) matrix via solvent casting from chloroform, yielding a transparent composite material (Figure S12a, see details in Supporting Information). Note that PMMA was selected as a matrix for its amorphous nature, which ensures optical transparency and minimal absorption in the relevant spectral range. The composite material was irradiated at 340 nm for 12 min—a duration defined based on prior kinetic studies—to induce dimerization and form **QD** directly in the solid-state. The reaction efficiency was then quantified by dissolving the material in chloroform and analyzing its the absorption spectra, confirming successful dimerization with a 95% conversion to **QD** (Figure S12b). PMMA-QD composite, loaded at 1 wt % was similarly prepared using a solvent casting process (see Supporting Information for details). The resulting transparent material was thermally treated at 190 °C for 90 min—a condition selected based on prior kinetic data to ensure rapid and quantitative cleavage. Although higher temperatures could accelerate the reaction, 190 °C was selected to prevent degradation of the PMMA matrix, which is reported to typically occur above approximately 200 °C.^55,56^ Under these conditions, UV–vis analysis confirmed the complete recovery of **QM** (Figure S13a,b). In comparison, a photoinduced reversion in the solid-state at 265 nm reached only 40% reversion due to the establishment of a photostationary state in which monomer and dimer coexist (Figure S14).^27^ These findings underscore the advantage of thermal activation in enabling complete and unambiguous switching, positioning it as a more robust and scalable approach for property modulation in bulk materials.

### Advanced Functional Materials

As a next step, the newly introduced reversible bonding/debonding chemistry was applied to establish photothermal reversible crosslinking within polymer networks. To ensure efficient photothermal activation and clear observation of mechanical changes, a low glass transition (T_*g*_), amorphous polymer matrix was deliberately selected to provide sufficient chain mobility and maximize light penetration by minimizing crystallinity. Accordingly, a methyl methacrylate quinolinone monomer (**QMMA**, Figure 5a) was synthesized following established protocols (see details in Supporting Information).^57^ **QMMA** was subsequently copolymerized with 2-ethylhexyl methacrylate (**EHMA**) via free radical polymerization at a 1:9 ratio, yielding a random copolymer, referred to as **PQM** (Figure 5a), for which gel permeation chromatography (GPC) analysis revealed a monomodal molecular weight distribution with a number-average molecular weight (*M*_*n*_) of 15.2 × 10^3^ g/mol (Figure S15a,b). The statistical incorporation of **QMMA** into the polymer backbone was confirmed by GPC coupled with a UV–vis detector, revealing an overlap of the refractive index (dRI) and UV signals at a retention time of 14.73 min. Furthermore, ^1^H NMR spectroscopy revealed the presence of distinct **QM** signals, confirming the integrity of the quinolinone moieties after polymerization and enabled the determination of the degree of functionalization, calculated to be 11.8 mol % (see molecular characterization and calculation in Supporting Information).^57^ Additionally, absorption spectrum of the polymer in acetonitrile displayed the characteristic bands of **QM** (Figure S16) suggesting the integrity of the responsive motif. Finally, thermal analysis indicated stability up to 275 °C (5 wt % mass loss) and DSC traces showed a low glass transition temperature of 20.8 °C (Figure S17a–c), consistent with a soft material.

**Figure 5:**
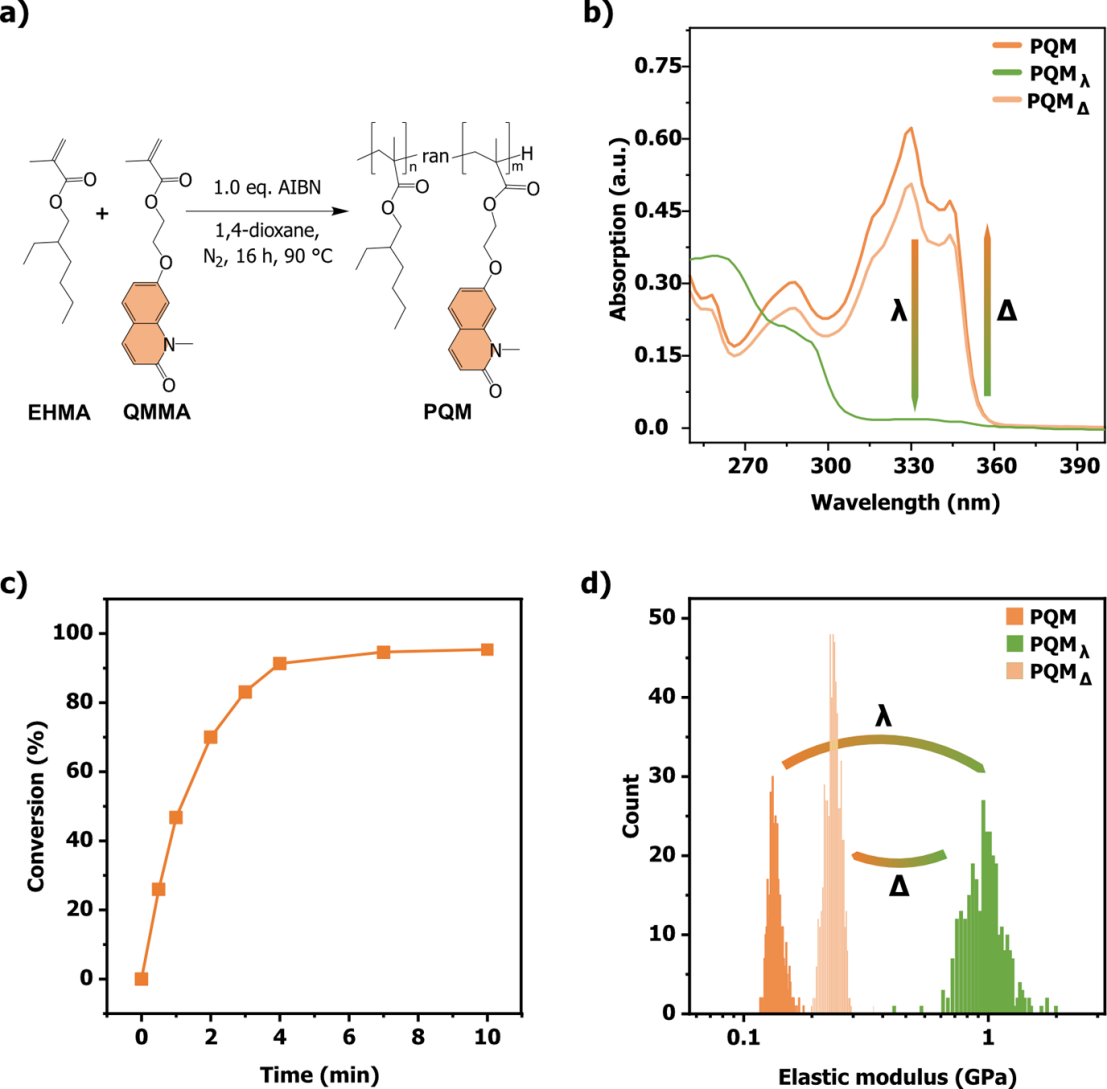
a) Reaction scheme for the synthesis of a **PQM** via copolymerization of 2-ethylhexyl methacrylate (**EHMA**) and the methyl methacrylate quinolinone monomer (**QMMA**). b) UV–vis absorption spectra of **PQM** film (dark orange), **PQM** after 12 min irradiation at 340 nm under oxygen free conditions (green) and irradiated **PQM** subsequently heated at 190 °C for 90 min. c) Kinetics of photodimerization, showing the conversion during the cross-linking of the **PQM** film. d) Elastic modulus for **PQM** (orange), **PQM** after light exposure­( λ , light orange) and **PQM** after light exposure (λ) and heating to 190 °C for 90 min (Δ, green), measured by atomic force microscopy (AFM)-based indentation.

Following complete characterization, **PQM** was processed into thin films to evaluate its photoresponsive behavior and functional performance. A solution of **PQM** in chloroform (50 μL of a 100 mg/mL solution) was spin-coated onto quartz slides and subsequently annealed overnight at 50 °C under reduced pressure (<200 mbar), producing a ∼4 μm thick, sticky and transparent film (Figure S17a). The coating was then irradiated with LEDs at λ = 340 nm (power: 55 mW, details in Supporting Information) for 12 min at ambient temperature under oxygen free conditions, initiating the photodimerization of the quinolinone groups and forming a crosslinked network. Dimerization was monitored in the solid-state using UV–vis spectroscopy and evidenced by the disappearance of the **QM** monomer absorption alongside the emergence of the **QD** absorption bands upon irradiation (Figures 5b, S18). Kinetic analysis of the photocycloaddition process, performed at intervals of 0.5 to 3 min, revealed a 95% conversion of quinolinones into their dimers after 7 min of irradiation, thus achieving maximum crosslinking density in **PQM** (Figure 5c, see details in the Supporting Information).

The gel point of the photocrosslinked **PQM** was determined by monitoring the changes in absorption of the irradiated coating dissolved in chloroform over time (see details in Supporting Information). The gel point was defined as the intersection of two linear regressions: one representing the initial rapid decrease in chloroform-soluble species, and the other corresponding to the plateau phase, where solubility remained constant (Figure S19). The gel point was reached after 130 s of irradiation, corresponding to approximately 72% conversion based on kinetic analysis. This transition signifies the formation of an infinite polymer network, further corroborating the complete conversion of quinolinones into dimers and the resulting enhancement of the materials’ structural integrity.

Atomic force microscopy (AFM) indentation experiments, performed with a 50 nm-radius spherical cantilever tip, were employed to probe surface mechanical changes, specifically the elastic (storage) modulus (Figure S20, see details in Supporting Information).^65^ It is important to note that AFM-based indentation provides a localized mechanical information over areas of approximately 100 μm^2^ per force map and does not account for viscous properties. Nevertheless, the elastic moduli obtained serve as a representative metric for comparing samples, based on up to six force maps per material. Pristine **PQM** exhibited an elastic modulus of 133 ± 10 MPa, significantly higher than the 42 ± 2 MPa measured for poly­(ethylhexyl methacrylate) (**PEHMA**), a reference polymer without quinolinone groups (Figure 5d, see structure details in Supporting Information). This observed mechanical enhancement is attributed to the incorporation of quinolinone moieties as pendant groups, whose aromatic nature enables π–π stacking interactions, that locally induce crystalline domains within the otherwise amorphous polymer matrix. Such localized ordering contributes to the 3-fold increase in the elastic modulus. Upon irradiation (12 min, λ = 340 nm) **PQM** showed a marked increase in elastic modulus to 969 ± 226 MPa (Figure 5d), reflecting a 7-fold enhancement consistent with the crosslinked network formation. In contrast, **PEHMA** lacking **QM** groups exhibited no change in elastic modulus upon identical irradiation conditions (Figure S21).

The reversibility of the crosslinked **PQM** network was demonstrated by heating the bulk material at 190 °C for 90 min, conditions previously established to induce thermal cleavage of quinolinone dimers. UV–vis spectroscopy confirmed the regeneration of the linear polymer, evidenced by recovery of the monomer absorption and the absence of residual cross-linked species (Figure 5b). This finding was further corroborated by ^1^H NMR spectroscopy showing the recovery of the linear polymer structure (Figure S22).

AFM analysis of the thermally treated sample revealed a reduction in elastic modulus to 233 ± 24 MPa (Figure 5d), consistent with the loss of covalent crosslinks. However, the modulus remained slightly elevated compared to the original uncrosslinked **PQM** (133 MPa), indicating incomplete recovery of the mechanical properties. The observed residual stiffness cannot be attributed to significant unbroken covalent crosslinks, as both UV–vis and ^1^H NMR spectroscopy confirm full cleavage of the quinolinone dimers. However, the presence of minor residual covalent crosslinks or degradation products below the detection limits of these techniques cannot be entirely ruled out and may contribute to the remaining stiffness.

To further investigate the origin of this residual stiffness, HPLC analyses were performed on solid-state blends of **QM** in synthesized **PEHMA** after 12 min of irradiation at 340 nm, under oxygen-free conditions (see Supporting Information). The chromatograms revealed two distinct species, including the expected anti head-to-head isomer and a second, unidentified photoproduct (Figure S23). Upon thermal treatment, both species fully reverted to monomeric **QM**, confirming that the observed mechanical irreversibility is not due to the formation of noncleavable or irreversibly crosslinked species. It is important to note that analogous experiments conducted under ambient (oxygen-containing) conditions resulted in the absence of **QM** signals by HPLC (Figure S24), indicating that the presence of oxygen triggers side reactions such as degradation or covalent attachment of the chromophores to the polymer matrix. These irreversible modifications compromise reversibility and highlight the critical importance of oxygen-free conditions for maintaining photocyclability and enabling network recovery. Under inert conditions, the limited mechanical recovery is therefore not attributed to incomplete dimer cleavage or oxidative side reactions. Rather, the residual stiffness likely stems from subtle degradation processes intrinsic to the polymer matrix, such as low levels of thermally or photochemically induced damage. These changes appear to be minor after the first photothermal cycle and are not detectable by spectroscopy or HPLC (Figure S22). However, upon subjecting the material to a second full cycle, alterations in the NMR spectra become evident, indicating that irreversible chemical modifications accumulate with repeated processing (Figure S25). This is further supported by observed variations in the **PEHMA** matrix following processing (Figure S26). Taken together, the results confirm that the system supports chemically reversible dimer cleavage and enables recyclability. However, its long-term mechanical reprocessability is limited by cumulative matrix degradation. These initially minor irreversible changes, likely stemming from low levels of thermal or photochemical side reactions, remain undetectable after the first cycle but become evident upon repeated activation, as shown by changes in the NMR spectra. Improving durability may therefore require adjusting irradiation and thermal parameters or adopting more inert matrix systems to suppress such effects.

Despite these limitations, recyclability was successfully demonstrated when decrosslinked films subjected to a second photochemical treatment exhibited renewed dimer formation, as evidenced by a further decrease in the monomer UV absorption spectrum (Figure S27a,b). This demonstration confirms that the network retains its photoresponsive character and can be recrosslinked after use, emphasizing its promise as a reversible material platform.

Beyond reversibility, the multiresponsive behavior of quinolinone—its ability to undergo controlled changes in mechanical properties under different stimuli—offers opportunities to design advanced functional materials. These features can be leveraged to develop coatings or adhesives capable of on-demand debonding and eventual recyclability. To illustrate this concept, **PQM** was applied as a coating on a glass substrate and used as an on-demand adhesive, with properties modulated by selective external stimuli. Upon irradiation at 340 nm for 12 min, the coating transitioned from soft, low T_*g*_ materials to a solid, nontacky film (Figures 6a,b,d), attributed to crosslinking that raises T_*g*_ above room temperature and imparts mechanical robustness. Interestingly, subsequent irradiation at 265 nm, targeting the quinolinone dimer reversion absorption, induced only partial decrosslinking (∼40%), consistent with the photostationary state typically observed during depolymerization in bulk (Figure 6c,d).^27^ Despite incomplete chemical reversion, this partial decrosslinking was sufficient to lower the T_*g*_ below room temperature, restoring tackiness. This was demonstrated by successfully gluing a small “pin” to the surface, evidencing regained adhesive function while retaining a crosslinked network (Figure 6c,d,e). Full reversibility was demonstrated by subjecting the coated substrate to thermal treatment at 190 °C, which triggered complete decross-linking and allowed the polymer film to be cleanly removed from the surface (Figure 6d–f). A demonstration of this functional and recyclable coating is provided in the Supporting Information Movie.

**Figure 6:**
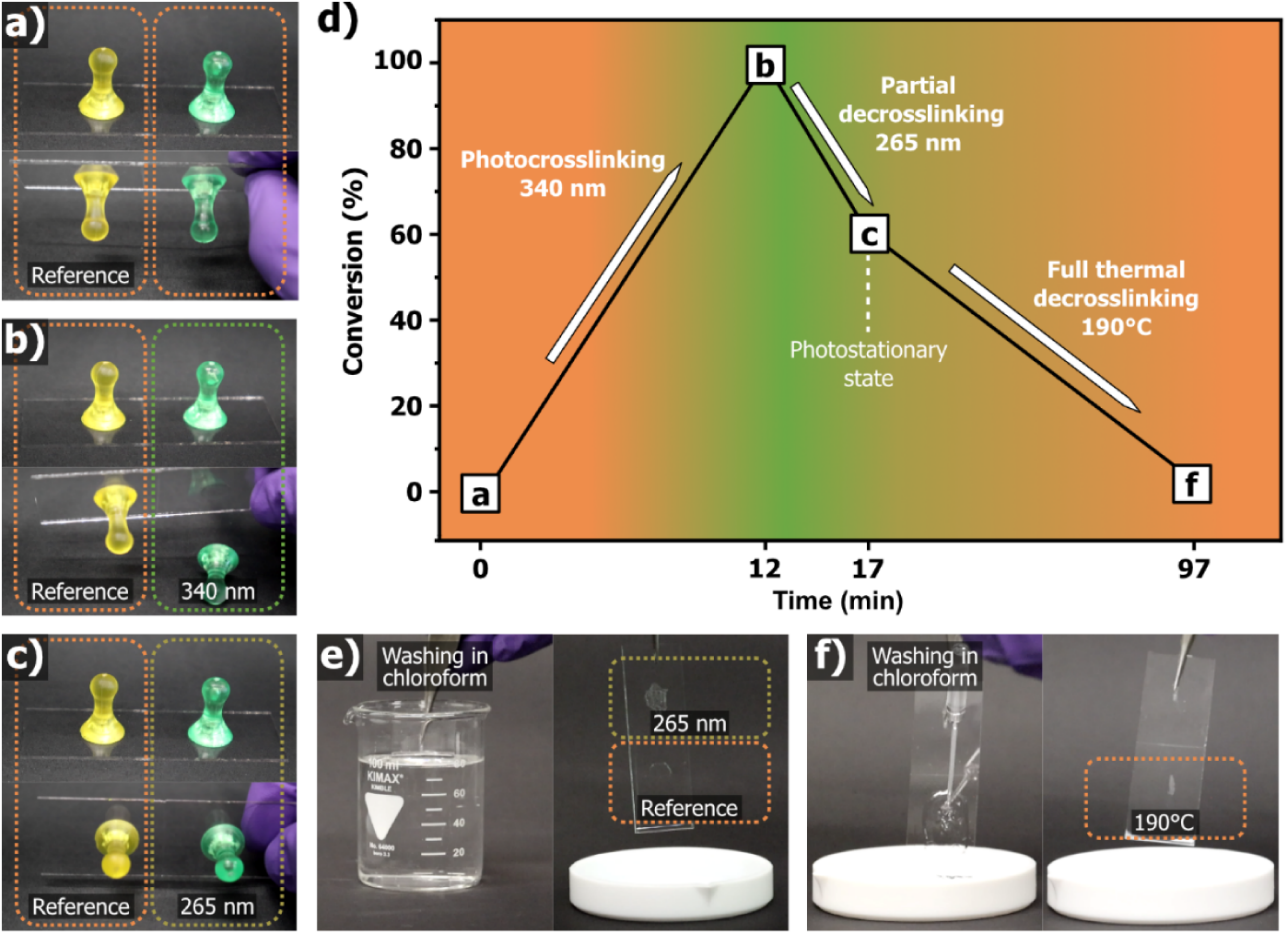
a–c) Photographs of a test object (a plastic pin) adhered to a glass substrate using **PQM** coatings under different light treatments. In each panel, the left image shows the pawn adhered using a pristine (non-irradiated) **PQM** coating, while the right image shows the same test after light treatment: (a) no irradiation (control), (b) irradiation at 340 nm for 12 min, (c) sequential irradiation at 340 and 265 nm. d) Summary of conversions for photodimerization (crosslinking) and partial photocleavage (decrosslinking) under the respective conditions shown in (a–c), with corresponding processing times. e) Chloroform rinsing of samples in (c): left, prior to rinsing; right, after rinsing, showing residual crosslinked coating. f) Chloroform rinsing of the irradiated and thermally treated **PQM** coating: left, before wash; right, after wash, showing complete removal from the glass.

To further demonstrate the functional behavior of the quinolinone-based material, creep tests were performed on single lap joints where **PQM** films bonded two glass slides under controlled loading conditions (Figure S28). When subjected to a constant load of 5.9 N, the sample exhibited creep behavior with a displacement of 0.7 mm over 9.5 min. In comparison, after irradiation at 340 nm for 12 min, the sample responded with immediate elastic deformation and negligible time-dependent displacement, indicating a predominantly elastic behavior with minimal viscous effect. This shift is consistent with the formation of covalent crosslinks and is further confirmed by UV–vis analysis of the solubilized sample, which revealed a significant decrease in the absorption band associated with **PQM**. Following thermal treatment of the irradiated film, the creep profile changed noticeably, exhibiting a larger displacement (1.6 mm in 38 min) under identical loading conditions. This increased deformation reflects a recovery of molecular mobility caused by disruption of the crosslinked network. As a result, the material softens, but still retains some residual elasticity. Consistently, the UV–vis spectrum of the solubilized film showed a near-complete restoration of the original **PQM** absorption band associated with the quinolinone monomer, confirming that thermal treatment efficiently cleaves the dimers and enables reversible decrosslinking of the network. Together, this stimulus-responsive behavior exemplifies the potential of quinolinone-based materials for recyclable adhesives and reconfigurable coatings, where mechanical integrity and adhesion can be modulated on demand.

## Conclusion

In summary, quinolinones and their conjugated dimers showcase remarkable versatility as thermally and photochemically responsive systems. At the molecular level, these dimers exhibit exceptional thermal stability, withstanding temperatures up to 170 °C, which ensures compatibility with diverse polymer processing conditions without inducing degradation. Beyond this threshold, the dimers undergo thermally induced cycloreversion, enabling precise and quantitative cleavage. Notably, this is the first demonstration of such behavior in a coumarin dimer derivative, which typically displays high thermal resistance. By increasing the temperature, the cleavage time can be significantly reduced, highlighting the system’s controllability and tunability. Once cleaved, the molecular building blocks can be reassembled through photochemical processes, enabling redimerization and facilitating reversible cyclability across at least three cycles without efficiency loss. This seamless transition between thermal cleavage and photoreversible bonding underscores the robust and multiresponsive nature of quinolinones, opening pathways for the design of advanced reversible and circular systems.

On the macroscale, the system demonstrates the ability to create crosslinked networks that can be decrosslinked and reformed on demand. This responsivity, combined with a high activation range (up to 300 °C for depolymerization), ensures broad applicability in polymer processing and high-temperature applications. Importantly, this photothermally reversible framework addresses a critical limitation of fully photoprocessed systems at the material level, where photostationary states hinder complete depolymerization. By combining thermal and photoinduced transformations, this system achieves near- full depolymerization, offering an effective alternative for dynamic material reconfiguration. Furthermore, this system also has the ability to precisely tune processing times and cross-linking levels introduces an orthogonal framework for designing innovative materials, such as debondable adhesives, functional coatings, and depolymerization strategies. Overall, the unique coupling of thermal and photoinduced transformations in quinolinones offers a powerful platform for developing advanced, sustainable, and reconfigurable materials. This system sets a precedent for designing versatile, robust, and efficient reversible chemical systems, paving the way for future breakthroughs in materials science.

## Supplementary Material





## Notes

Supplementary Figures S1–S28 and a comprehensive account of all experimental details, including synthetic procedures, analytical data, sample preparation, and mechanical measurements description (PDF)
A supporting movie demonstrating the functional and recyclable coating (MP4)
