# Supplementary material for: Orthogonal and Multiresponsive Quinolinone Systems for Reversible and Recyclable Polymer Networks
Source: J Am Chem Soc. 2025 Aug 27;147(36):32831–40. doi: 10.1021/jacs.5c08976 (PMC12426877; doi:10.1021/jacs.5c08976)
Supplement: Supplementary file 1 [file ja5c08976_si_001.pdf]

SUPPORTING INFORMATION

for

Orthogonal and Multiresponsive Quinolinone Systems for Reversible and Recyclable Polymer Networks

*Claas-Hendrik Stamp†1, Annalena Groß†2, Aitana Beato1, Bizan N. Balzer1,3,4, Céline Calvino1,2**

[1]C.-H. Stamp, A. Beato, B. N. Balzer, C. Calvino
Albert Ludwig University of Freiburg – Cluster of Excellence *liv*MatS, Georges-Köhler-Allee 105, D-79110 Freiburg, Germany
E-mail: Celine.calvino@livmats.uni-freiburg.de

[2]A. Groß, C. Calvino
Albert Ludwig University of Freiburg – Department of Microsystems Engineering (IMTEK), Georges-Köhler-Allee 102, D-79110 Freiburg, Germany
E-mail: Celine.calvino@livmats.uni-freiburg.de

[3] B. N. Balzer
Albert Ludwig University of Freiburg – Institute of Physical Chemistry, Albertstr. 21, D-79104 Freiburg, Germany

[4] B. N. Balzer
Albert Ludwig University of Freiburg – Freiburg Materials Research Center (FMF), Stefan-Meier-Str. 21, D-79104 Freiburg, Germany.

Table of Contents:

1 Supporting Data 2

2 Supporting Experimental Section 16

3 References 444

1 Supporting Data

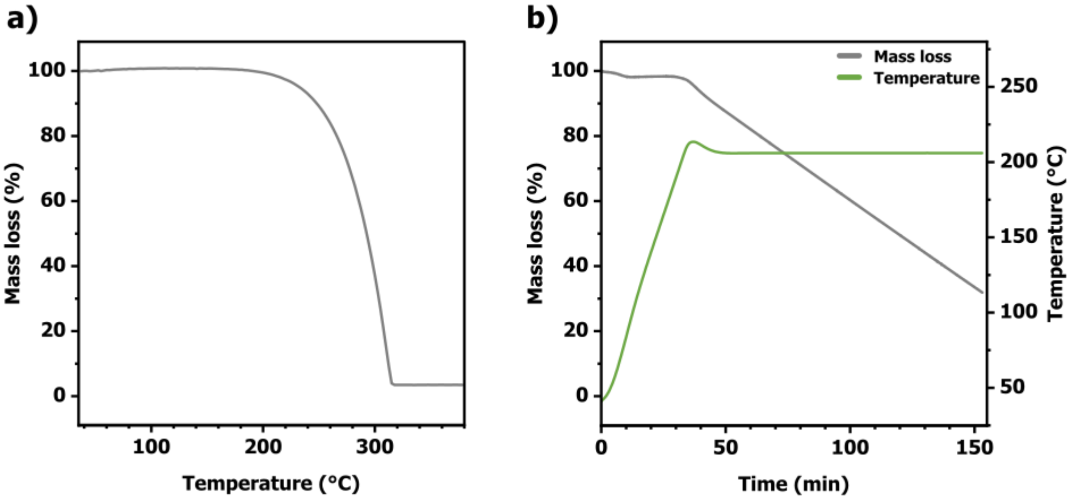

Figure S1. a) Thermogravimetric analysis (TGA) of **QD** recorded from 25 °C to 400 °C at a heating rate of 5 °C/min under a nitrogen atmosphere. **b)** TGA curve of **QM** subjected to a tempering process at 200 °C for 2 h. Grey curve represents the mass loss and the green curve the temperature profile applied over time.

Note: During the tempering process, a constant mass loss is observed due to the open TGA crucible, which allows the sublimation of the reverted dimer (**QM**), leading to continuous weight loss.

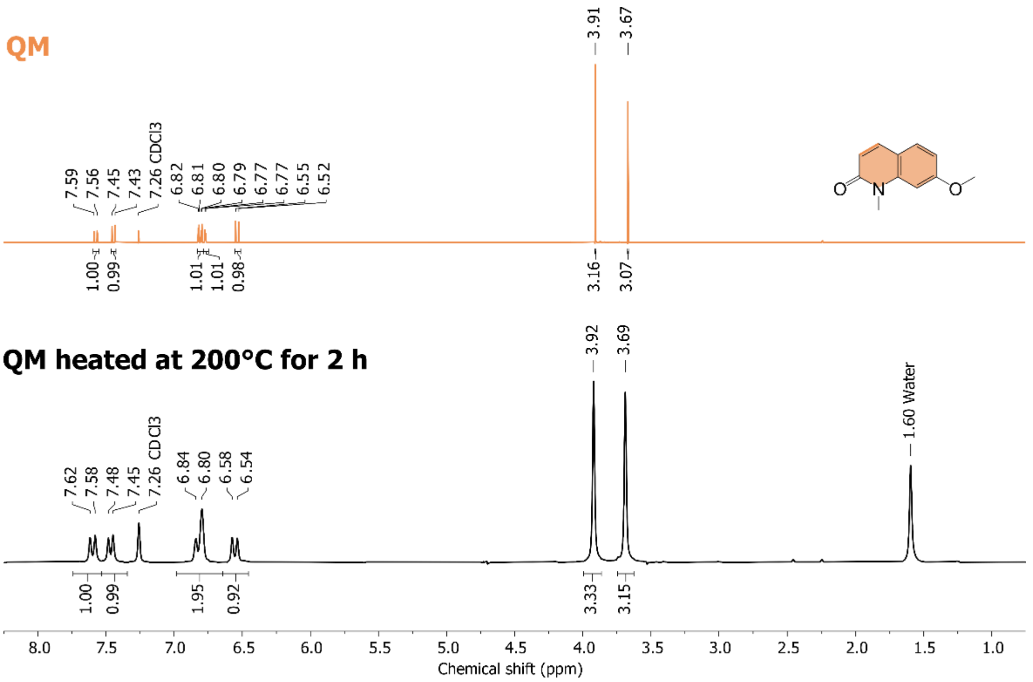

Figure S2. Comparison of the ^{1}H NMR spectra (400 MHz and 250 MHz, 297.2 K, $CDCl_3$) of **QM** after a tempering process at 200 °C for 2 h (black spectrum, corresponding to the TGA shown in Fig. S1b) with the reference spectrum for **QM** (orange).

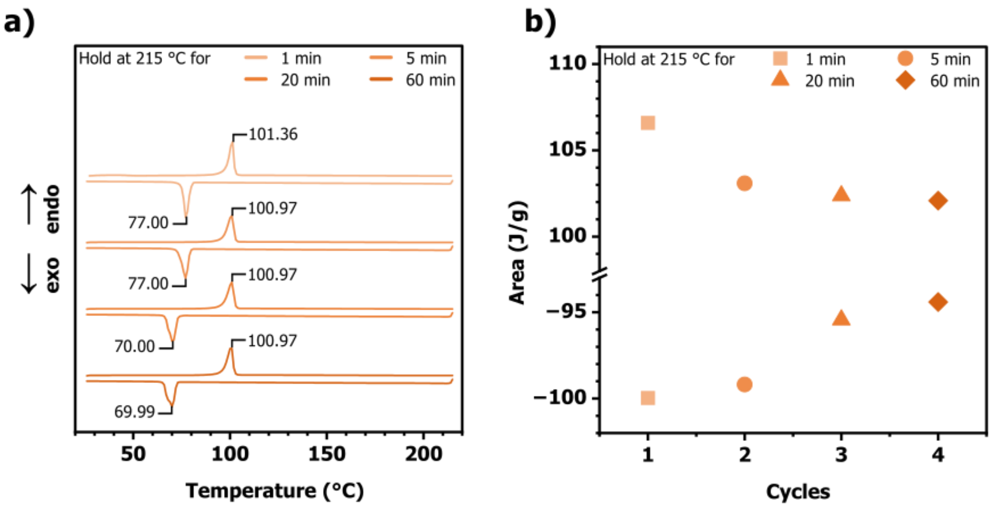

Figure S3. a) Differential scanning calorimetry (DSC) traces of **QM** over four heating and cooling cycles, with progressively increased isothermal segments at 215 °C, ranging from 1 to 60 min across the cycles. Note that the legend indicates the cumulative time of the applied isotherm. The traces were recorded from 25 °C to 215 °C at a heating rate of 5 °C/min and under a nitrogen atmosphere. **b)** Corresponding melting (endothermic transition at ca. 101 °C) and crystallization (exothermic transition ranging between ca. 70-77 °C) peak areas as a function of the isothermal time applied.

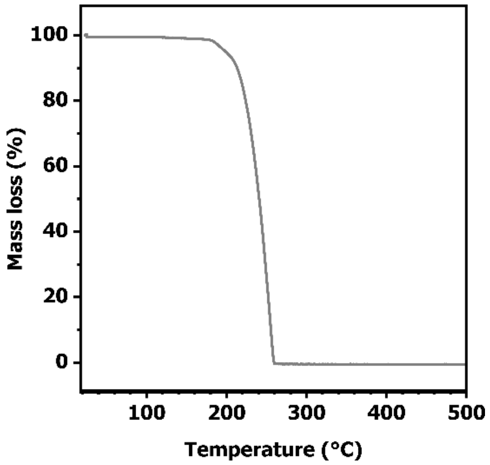

Figure S4. TGA curve of **QD** recorded from 25 °C to 500 °C at a heating rate of 5 °C/min, under a nitrogen atmosphere. The grey curve corresponds to the mass loss, while the green curve represents the derivative of the mass loss.

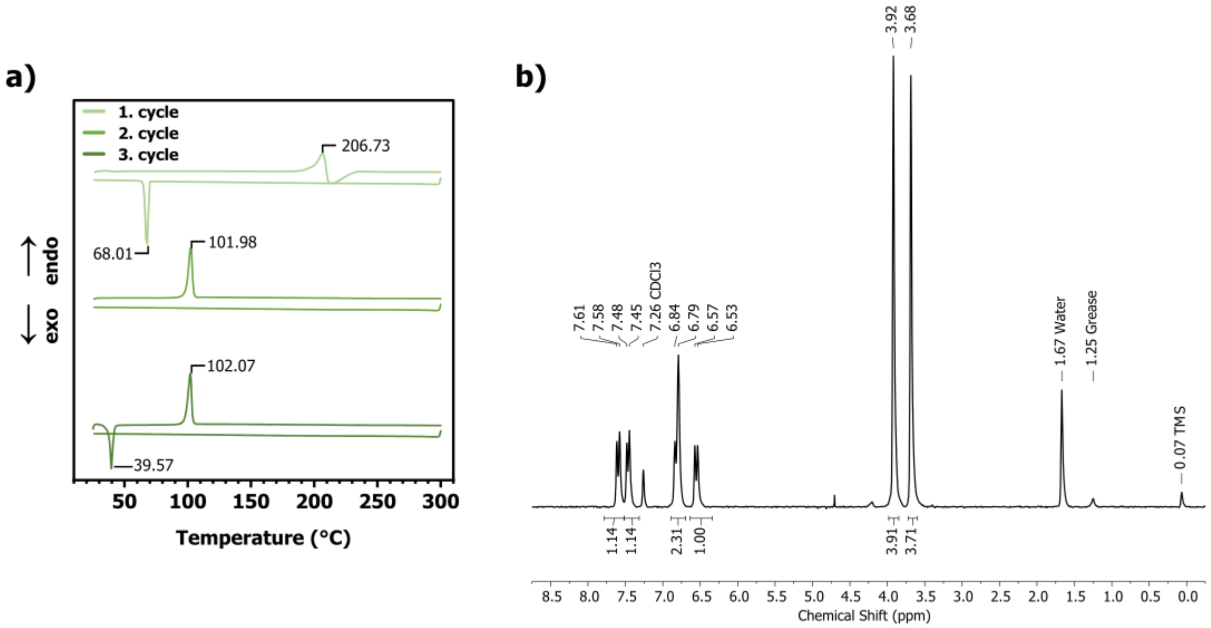

Figure S5. a) DSC traces of **QD** over three heating and cooling cycles, recorded from 25 °C to 300 °C at a heating rate of 5 °C/min, including a 5 min isothermal segment at 300 °C. **b)** ^{1}H NMR spectra (250 MHz, 297.2 K, $CDCl_3$) of the resulting product after the three DSC cycles.

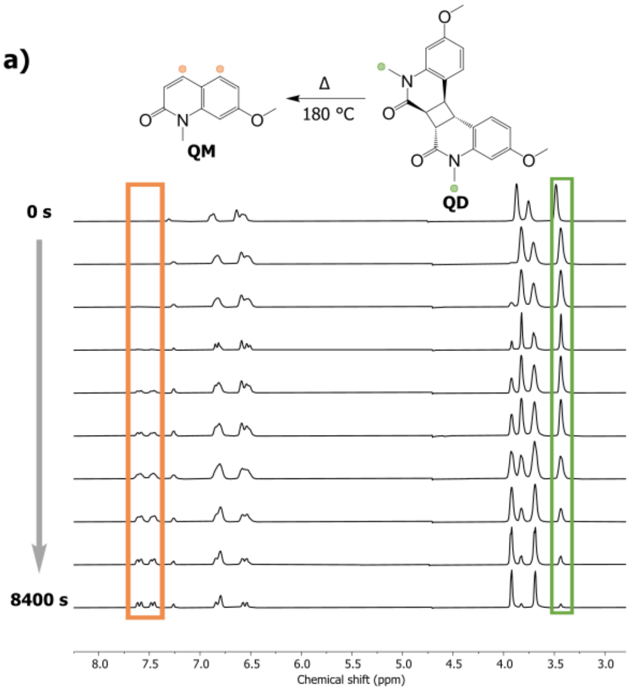

b)

Time (s)	QD: $-CH_3$	QM: *-H*
0	1	0.00
240	1	0.01
960	1	0.07
1680	1	0.13
2400	1	0.22
3000	1	0.33
4200	1	0.68
5400	1	1.5
6600	1	2.34
8400	1	5.75

Figure S6. a) ^{1}H NMR spectra (250 MHz, 297.2 K, $CDCl_3$) of **QD** heated at 180 °C for periods ranging from 0 to 8400 s. **b)** Table showing the ratio of the peak areas of the $-CH_3$ group of **QD** (3.53 to 3.23 ppm) and the aromatic signals of **QM** (7.74 to 7.37 ppm) at different heating times, based on the spectra presented in a).

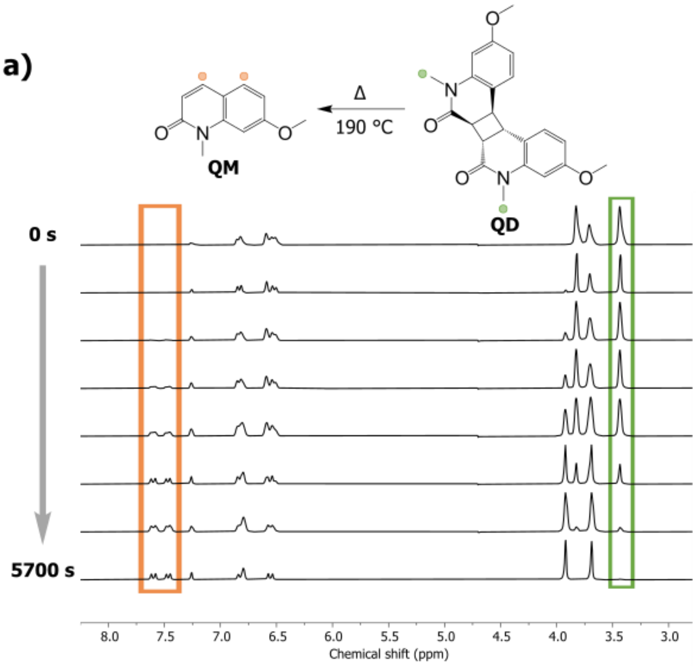

b)

Time (s)	QD: -CH_3	QM: -*H*
0	1	0.00
120	1	0.03
360	1	0.11
600	1	0.19
960	1	0.38
1560	1	1.07
2940	1	4.74
5700	1	25.39

Figure S7. a) ^{1}H NMR spectra (250 MHz, 297.2 K, $CDCl_3$) of **QD**, heated at 190 °C for periods ranging from 0 to 5700 s. **b)** Table showing the ratio of the peak areas of the –CH_3 group of **QD** (3.53 to 3.23 ppm) and the aromatic signals of **QM** (7.74 to 7.37 ppm) at different heating times, based on the spectra presented in a).

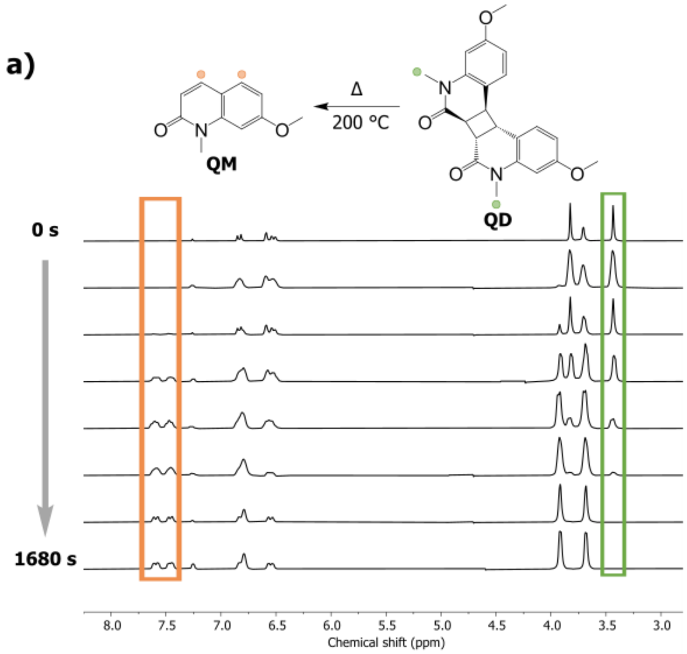

b)

Time (s)	QD: -CH_3	QM: -*H*
0	1	0.00
60	1	0.03
180	1	0.14
360	1	0.61
540	1	2.16
720	1	6.90
1140	1	33.7
1680	1	228.4

Figure S8. a) ^{1}H NMR spectra (250 MHz, 297.2 K, $CDCl_3$) of **QD**, heated at 200 °C for periods ranging from 0 to 1680 s. **b)** Table showing the ratio of the peak areas of the –CH_3 group of **QD** (3.53 to 3.23 ppm) and the aromatic signals of **QM** (7.74 to 7.37 ppm) at different heating times, based on the spectra presented in a).

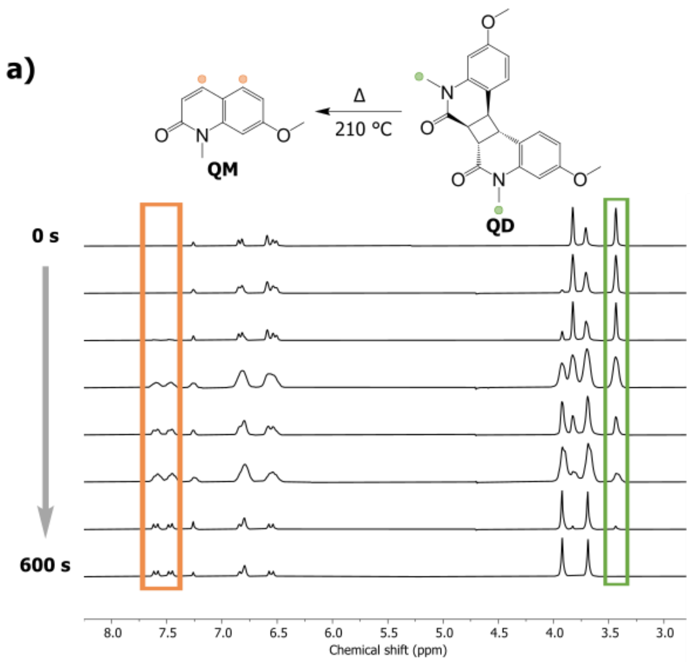

b)

Time (s)	QD: -*CH3*	QM: -*H*
0	1	0.00
30	1	0.04
60	1	0.13
120	1	0.45
180	1	1.06
240	1	2.38
360	1	5.83
600	1	33.48

Figure S9. a) ^{1}H NMR spectra (250 MHz, 297.2 K, $CDCl_3$) of **QD**, heated at 210 °C for periods ranging from 0 to 600 s. **b)** Table showing the ratio of the peak areas of the $-CH_3$ group of **QD** (3.53 to 3.23 ppm) and the aromatic signals of **QM** (7.74 to 7.37 ppm) at different heating times, based on the spectra presented in a).

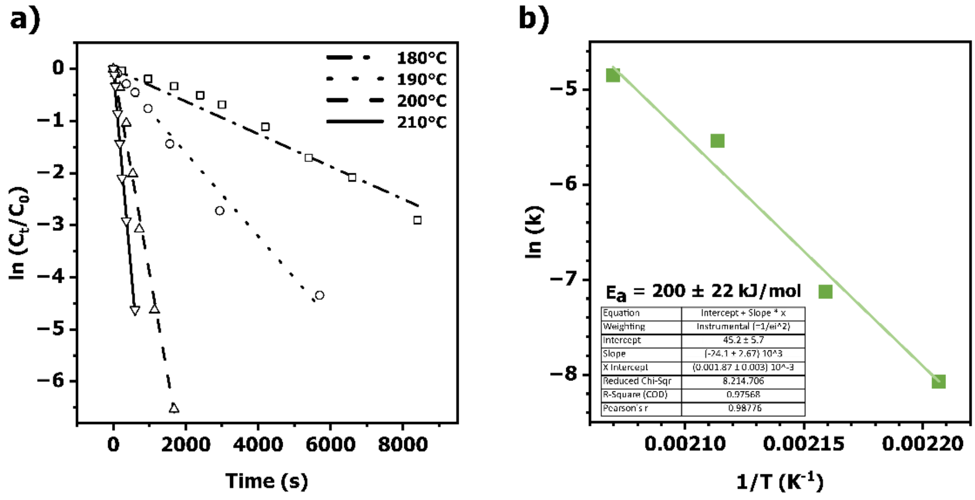

c)

	180°C	190°C	200°C	210°C
Equation	Intercept + Slope * x	Intercept + Slope * x	Intercept + Slope * x	Intercept + Slope * x
Intercept	0 ± --	0 ± --	0 ± --	0 ± --
Slope	(-3.1 ± 0.1) 10^-4	(-8.0 ± 0.3) 10^-4	(-39.2 ± 1.0) 10^-4	(-78.7 ± 1.6) 10^-4

Figure S10. a) Cycloreversion kinetics of **QD** upon heating at 180, 190, 200 and 210 °C (saturated solution = 500 µL + 100 µL in $CDCl_3$), derived from offline ^{1}H NMR monitoring. **b)** Arrhenius plot used to determine the activation energy for the thermal cycloreversion of **QD**. Calculated E_a = (200 ± 22) kJ/mol. **c)** Fitting parameters obtained from the individual pseudo first order kinetics depicted in a).

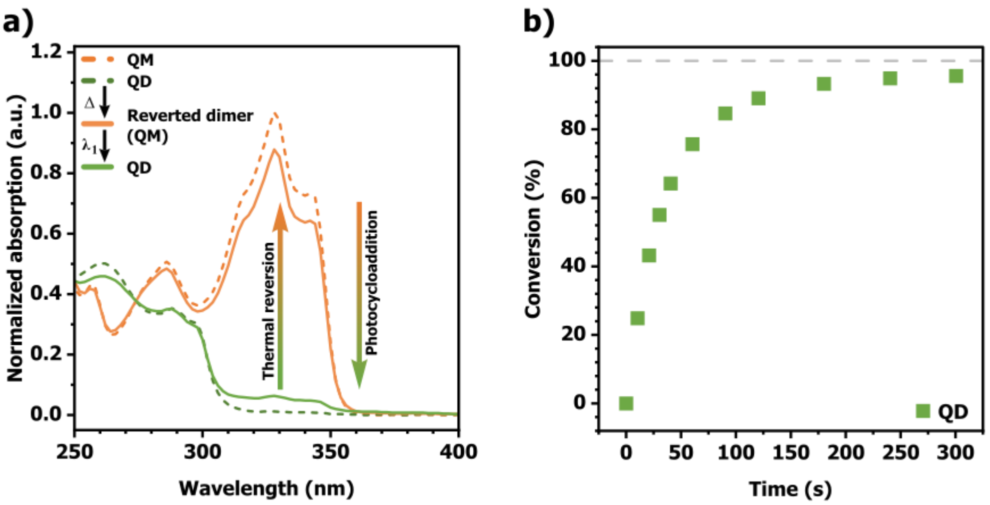

Figure S11. a) Ultraviolet-visible (UV-vis) absorption spectra of thermally reverted **QD** (orange bold spectra) after 5 min irradiation at 340 nm under nitrogen conditions (green bold spectra), **QM** (orange dashed line) and **QD** (green dashed line) included as references. **b)** Photocycloaddition conversion of the thermally reverted **QD**. The photoreactions were conducted in acetonitrile at c = 10^{-5} mol/L and irradiated with a LED photoreactor at a power of 33 mW. Photocycloaddition reactions were conducted in acetonitrile solutions at *c* = 6.5 10^{-5} mol/L.

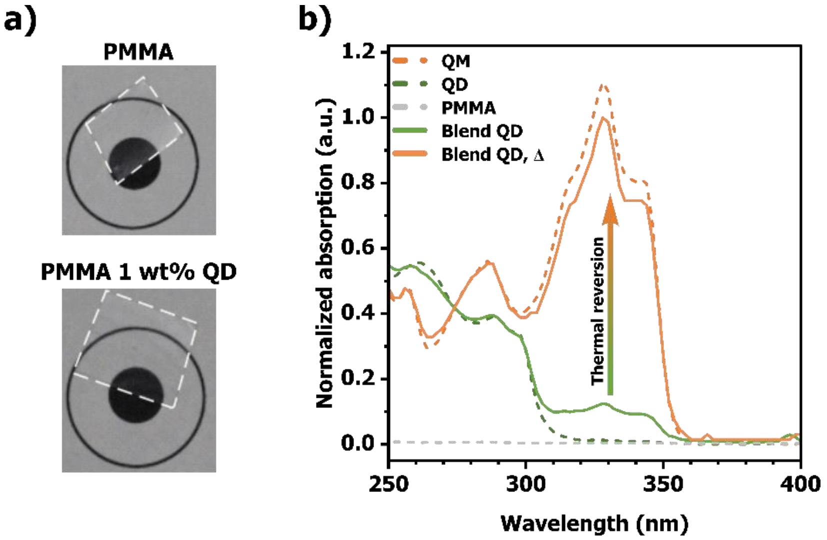

Figure S12. a) Photographs of PMMA blended with 1 wt % **QM**, before and after irradiation at 340 nm for 2 min **b)** UV-vis absorption spectra of PMMA-QM blends in solid state after 2 min irradiation at 340 nm under nitrogen conditions (black spectra), with **QM** (orange), **QD** (green) and neat PMMA included as references. The photoreaction was conducted in the solid state using a LED photoreactor at a power of 33 mW and **QM** absorption was measured in acetonitrile at *c* = 10^{-5} mol/L.

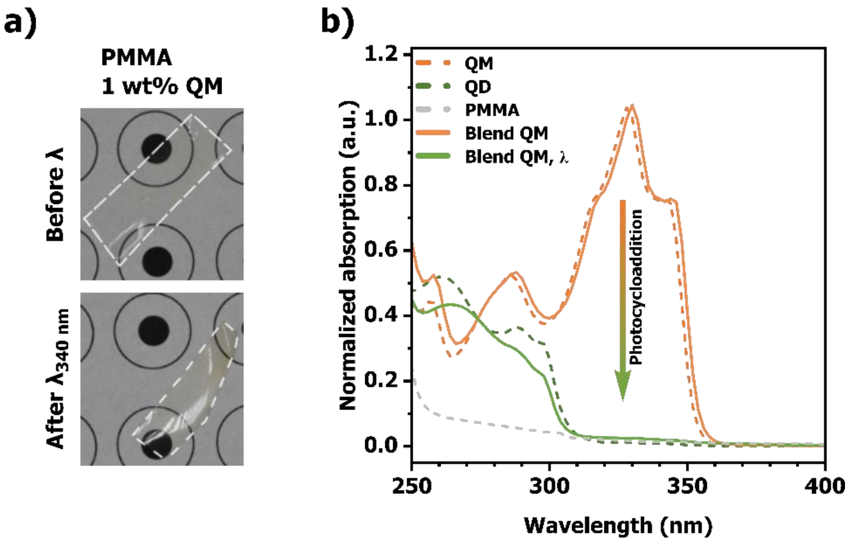

Figure S13. a) Photographs of neat PMMA and PMMA blended with 1 wt% **QD**. **b)** UV-vis absorption spectra of **PMMA-QD** blends after heating at 206°C for 60 min under a nitrogen atmosphere, with **QM** (orange), **QD** (green) and neat PMMA included as references. The tempering process was conducted in the solid state using a DSC and the heated material was dissolved in chloroform for measurement.

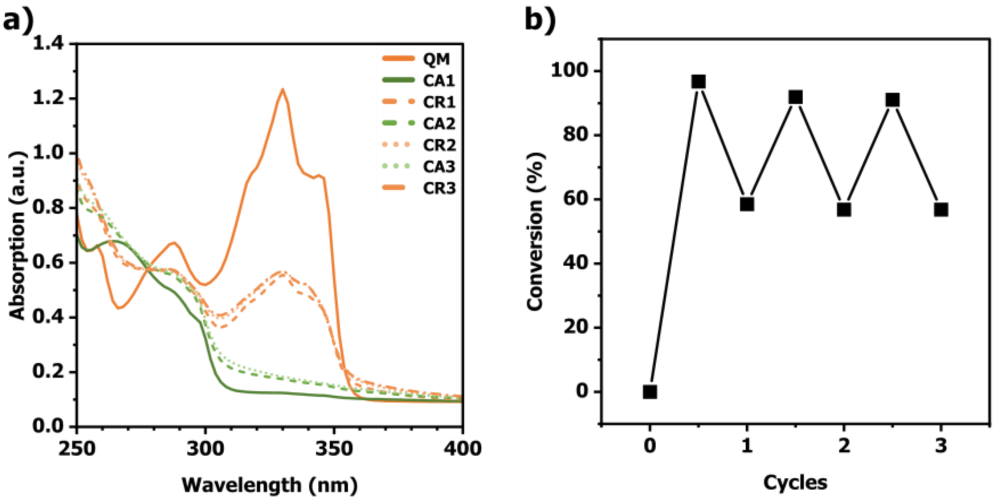

Figure S14. a) Sequential irradiation cycles of **PMMA-QM** (1 wt%), with cycloaddition (CA) induced by 340 nm irradiation for 2 min, followed by cycloreversion (**CR**) triggered by 265 nm irradiation for 12 min. The photoreactions were carried out under oxygen-free conditions using a LED photoreactor at power levels ranging from 22 to 33 mW. **b)** Conversion of the irradiation cycles calculated from (a).

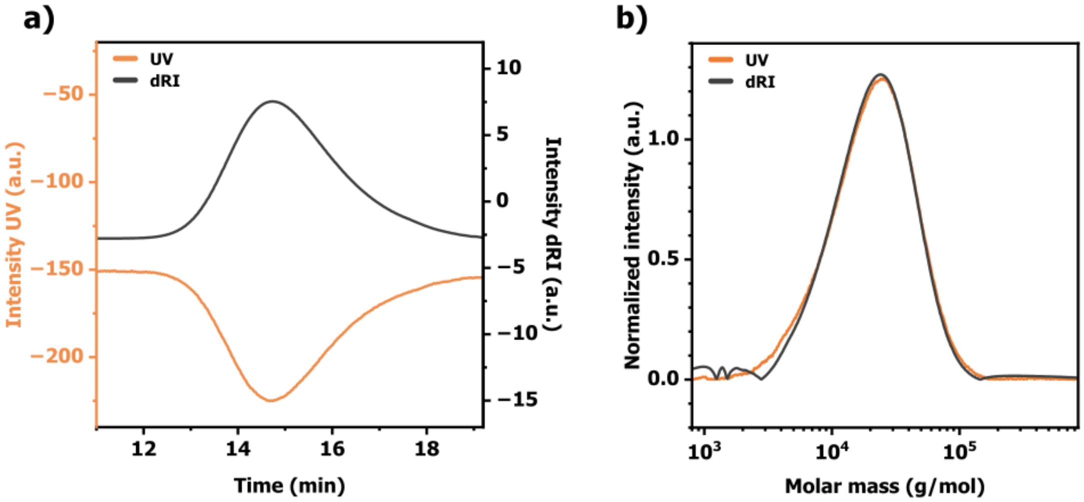

Figure S15. a) and b) Gel permeation chromatography (GPC) traces of **PQM** with the refractive index difference (dRI, black trace) and the UV detector signal (orange trace). The number-average molecular weight (M_n) was determined to be 15.2 10^3 g/mol at a retention time of 14.73 min, calibrated against a polystyrene standard column.

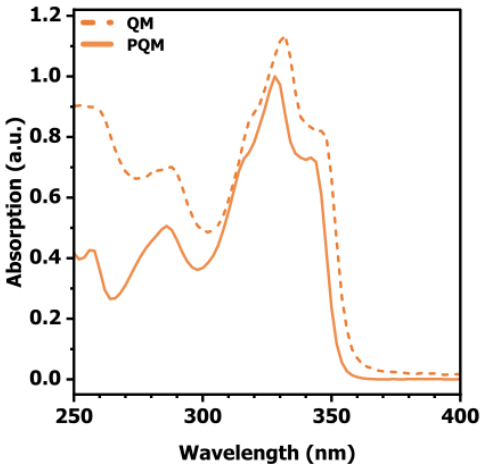

Figure S16. UV-vis absorption spectra of **PQM** in the solid state with **QM** (orange), included as references. **QM** absorption was measured in acetonitrile at c = 10^{-5} mol/L.

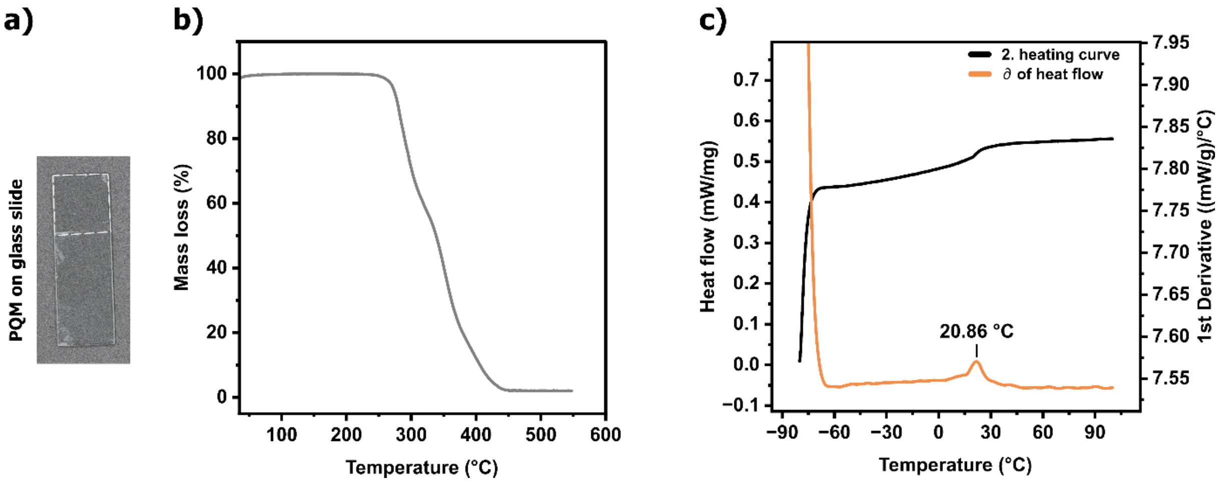

Figure S17. a) Photograph of **PQM** spin coated onto glass substrate. **b)** Corresponding TGA recorded from 30 °C to 550 °C at a heating rate of 5 °C/min under a nitrogen atmosphere and **c)** DSC analysis recorded from -80 °C to 100 °C at a heating rate of 5 °C/min. The glass transition temperature (T_g) was determined as the local maximum of the first derivative of the heat flow and was found to be 20.86 °C. The first derivative was smoothed using a Savitzky–Golay filter with a window size of 250 and a polynomial order of 2.

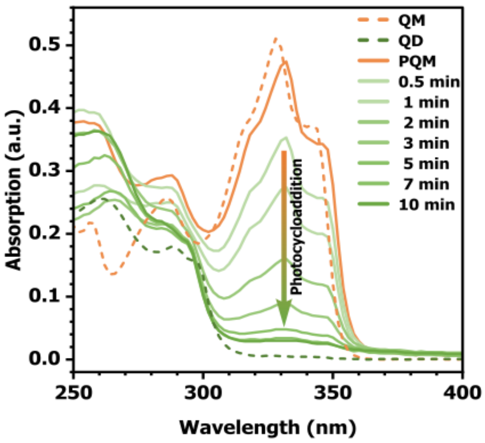

Figure S18. UV-vis absorption spectra of **PQM** after irradiation at 340 nm for 12 min and with **QM** (orange dashed line) and **QD** (green dashed line) included as references. The photoreactions were conducted under oxygen-free conditions using LEDs at a power level of 165 mW.

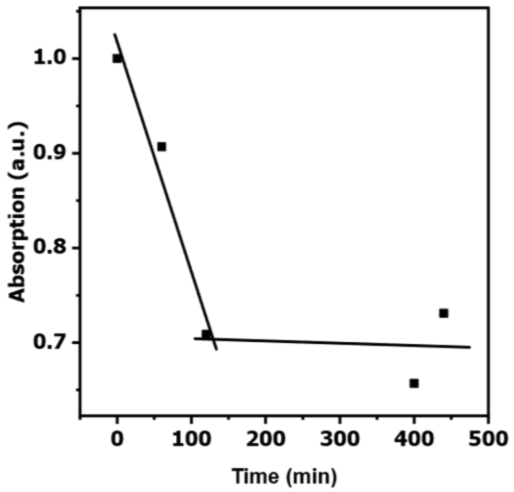

Figure S19. Determination of the gel point for photocrosslinked **PQM** films. Normalized UV–vis absorption at 328 nm of chloroform extracts from films irradiated at 340 nm for varying durations. The data were fitted with two linear regressions: one for the initial decrease (0–120 s) and one for the plateau region (120–480 s). The gel point is defined as the intersection of these two fitted lines

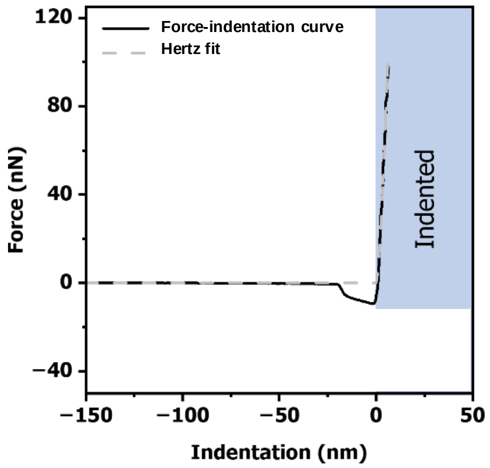

Figure S20. Elastic modulus detection by AFM-based force spectroscopy. An indentation curve (in black) using a spherical diamond-like carbon cantilever tip is taken and fitted by the Hertz model for the determination of the elastic modulus. The Hertz model-based fit is given by a gray broken line. The exemplarily data is shown for a **PQM λ** sample.

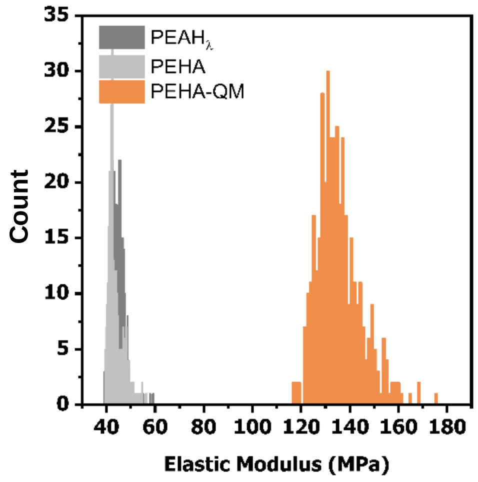

Figure S21. Elastic modulus for **PEHA**, **PEHA λ** and **PEHA-QM**. The Hertz model was applied as exemplarily shown in Fig. S20. A Gaussian fit to each histogram is used to obtain the mean value as the maximum of the Gaussian and the error as the width of the Gaussian: **PEHA**: (42 ± 2) MPa, **PEHA** I: (44 ± 4) MPa and **PEHA-QM**: (133 ± 10) MPa.

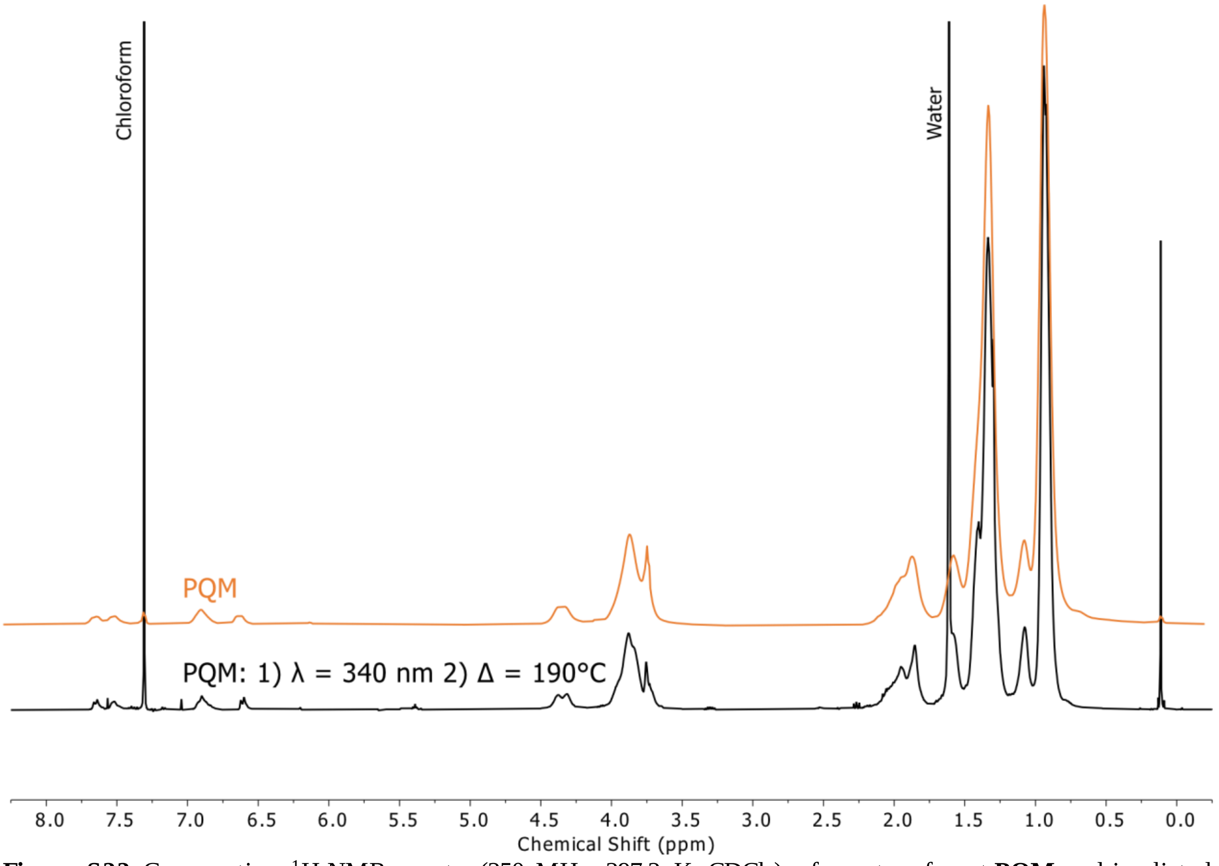

Figure S22. Comparative ^{1}H NMR spectra (250 MHz, 297.2 K, $CDCl_3$) of spectra of neat **PQM** and irradiated (λ = 340 nm) **PQM** film subsequently heated at 190 °C for 90 min. Full assigned spectra can be found in Section **2.3** .

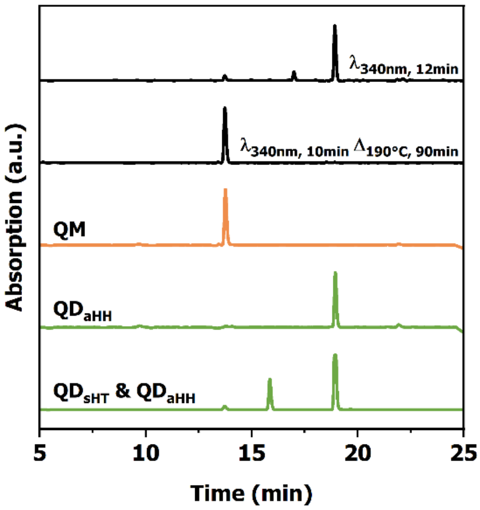

Figure S23. HPLC chromatograms of **PEHMA** blended with **QM** (1 wt% relative to **PEHMA**), recorded after irradiation (12 min, λ = 340 nm), and irradiated **QM** followed by subsequent heating (90 min, 190 °C) (black chromatograms). Both samples where irradiated and heated under oxygen free conditions. Chromatograms of **QM**, anti head-to-head dimer (**QD**), and a mixture of **QD** and syn head-to-tail dimer (**QD**$_{sHT}$) are provided for reference.

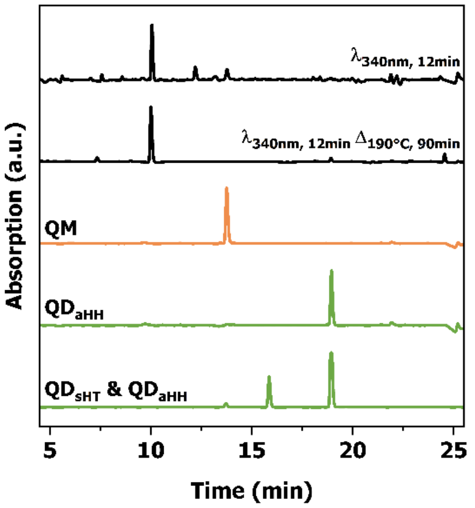

Figure S24. HPLC chromatograms of **PEHMA** blended with **QM** (1 wt% relative to **PEHMA**), recorded after irradiation (12 min, λ = 340 nm), and irradiated **QM** followed by subsequent heating (90 min, 190 °C). Both samples where irradiated or heated under atmospheric conditions. Chromatograms of **QM**, anti head-to-head dimer **QD**, and a mixture of **QD** and syn head-to-tail dimer (**QD**$_{sHT}$) are provided for reference.

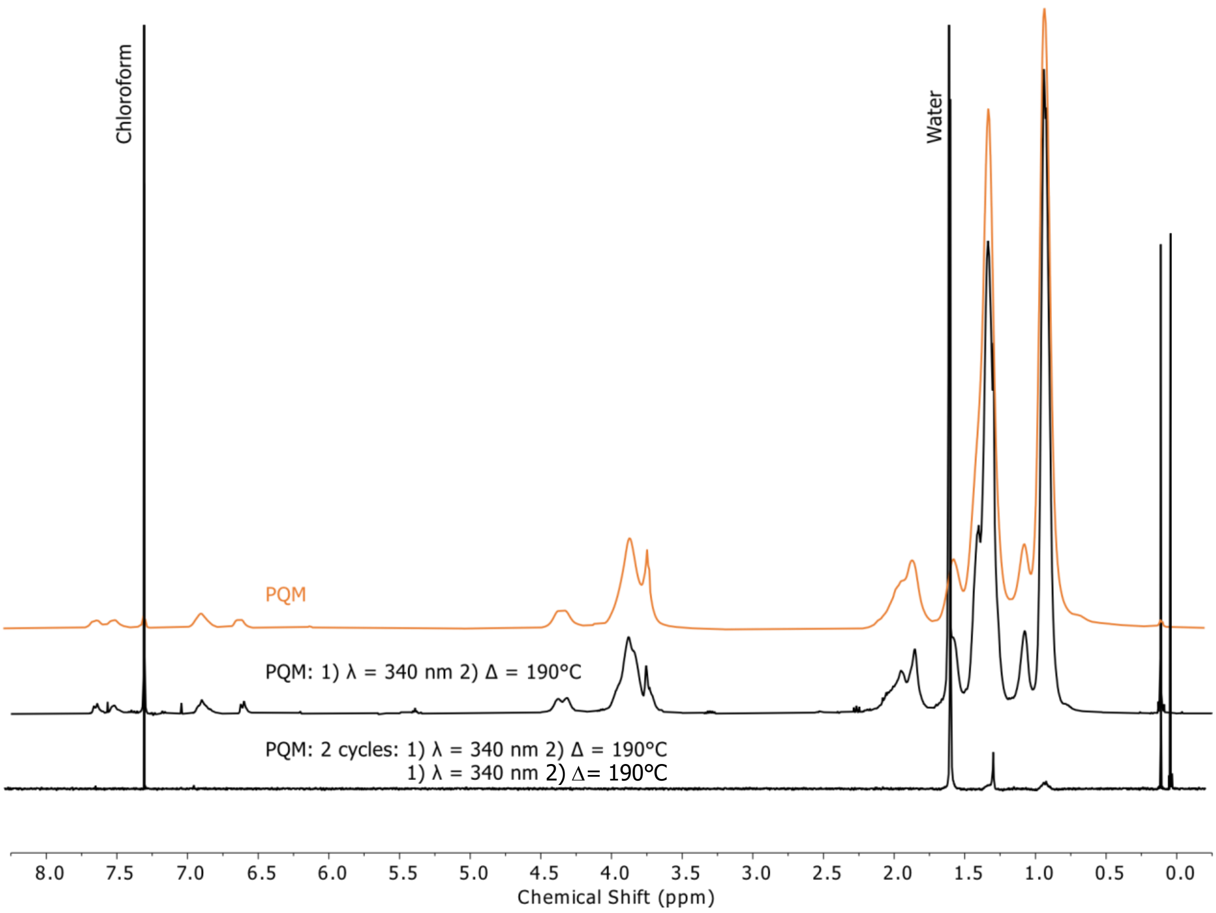

Figure S25. Comparative ^{1}H NMR spectra (250 MHz, 297.2 K, $CDCl_3$) of spectra of neat **PQM**, irradiated (λ = 340 nm, 12 min) **PQM** film subsequently heated at 190 °C for 90 min, and **PQM** after a second photo-thermo cycle.

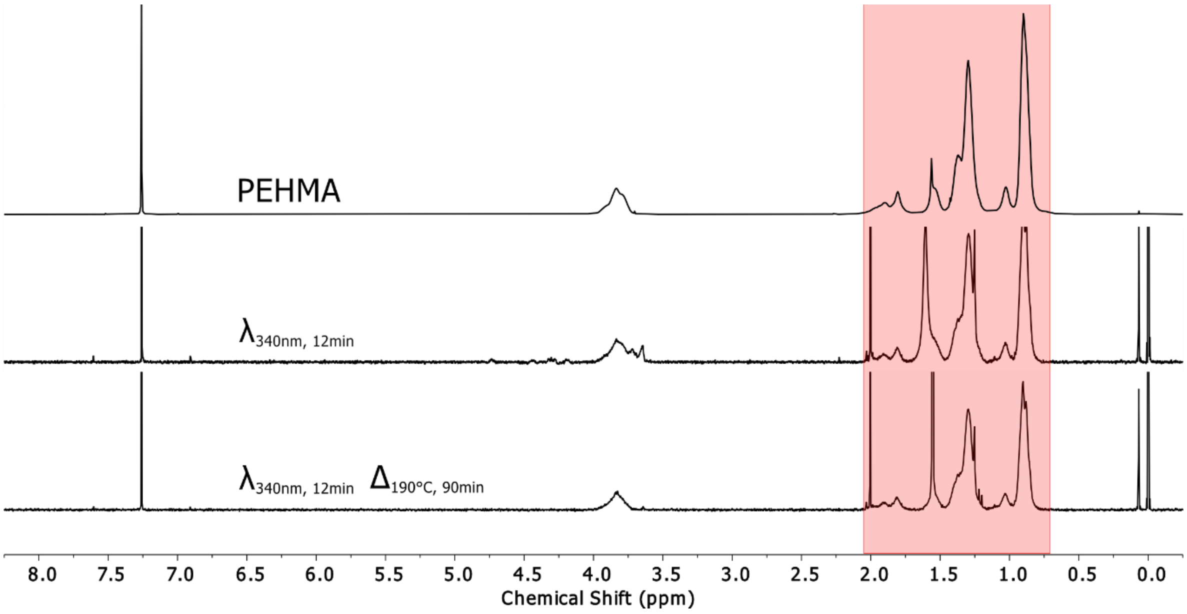

Figure S26. Comparative ^{1}H NMR spectra (250 MHz, 297.2 K, $CDCl_3$) of spectra of **PEHMA**, irradiated **PEHMA** film at 340 nm for 12 min, and irradiated **PEHMA** film subsequently heated at 190 °C for 90 min.

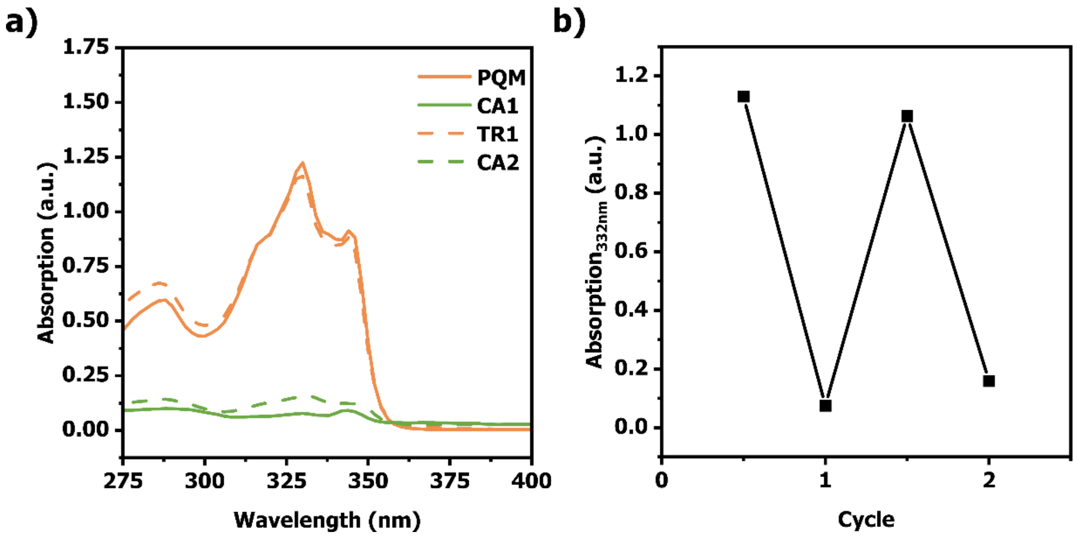

Figure S27. a) Sequential irradiation cycles of **PQM**, with cycloaddition (CA) induced by irradiation at 340 nm for 12 min, followed by thermal reversion (TR) triggered at 190 °C for 90 min. Both photo- and thermal reactions were carried out under oxygen-free conditions. **b)** Maximum absorption at 332 nm measured from the absorption spectra of each cycle illustrated in (a).

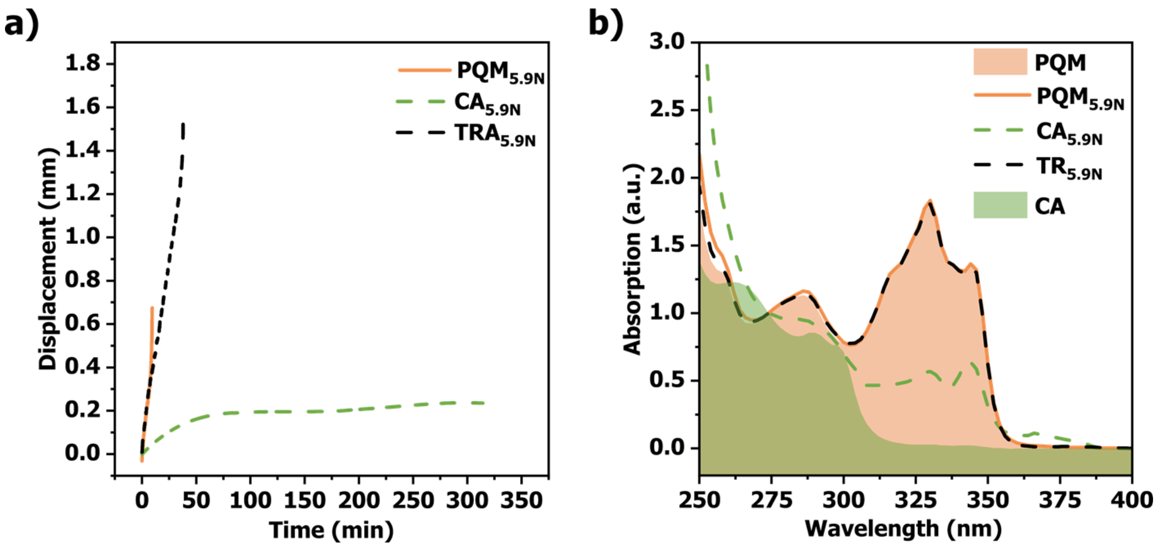

Figure S28. a) Creep displacement as a function of time for single lap joints made of two glass slides bonded with **PQM**. Curves correspond to: (i) pristine **PQM** under constant load (orange), (ii) **PQM** irradiated for 12 min at 340 nm (green), and (iii) irradiated **PQM** subsequently heated for 90 min at 190 °C. Displacement was tracked using subpixel Gaussian fitting. **b)** UV-vis absorption spectra of the PQM samples after creep testing shown in (a). Spectra were recorded from samples dissolved in chloroform following mechanical testing.

2 Supporting Experimental Section

2.1 Materials and Instrumentation

Materials. All reagents and solvents were used without further purification, if not specified otherwise. Acetone (>99 %), acetonitrile (ACN) (>99.5 %), chloroform-*d* (99.8 %) ($CDCl_3$), cyclohexane (>99.5 %) (CyH), dichloromethane (>99.5 %), dimethylsulfoxide-d_6 (99.8 %) (DMSO-d_6), 1,4-dioxane (>99,5 %), ethyl acetate (>99 %) (EtOAc), methanol (>99 %) (MeOH), N,N-dimethylformamide (DMF) (>99.5 %), silica gel 60 (0.03 - 0.2 mm) and, magnesium sulfate hydrate ($MgSO_4$) (>99 %) were purchased from Carl Roth. Dichloromethane and DMF were dried over pre-dried $MgSO_4$ (heated at 70 °C), directly distilled following the drying process, and stored over 4 Å molecular sieves. Pyridine hydrochloride (98 %), sodium hydride (60 % dispersion in paraffin wax), 2-ethylhexyl methacrylate (98 %, purified over a short column of basic aluminium oxide), methacryloyl chloride (97 %), triethylamine (TEA) (99.5 %) were purchased from Sigma Aldrich. Anhydrous potassium carbonate (K_2CO_3) (99 %), iodomethane (99 %) were purchased from Thermofisher. Basic aluminium oxide, chloroform (>99 %) were purchased from VWR. 2,2'-azobis(2-methylpropionitrile) (AIBN) (>98 %) was purchased from Fluka and recrystallized from acetone before use. 2-bromoethanol (>95 %) was purchased from TCI. 2,7-dihydroquinoline (iminol form of 7-hydroxyquinolin-2(1H)-one) was purchased from BLDPharm. L-ascorbic acid was purchased from Mivolis and Poly(methyl methacrylate) ($M_w = 550 \cdot 10^3$ g/mol) from abcr.

Nuclear magnetic resonance (NMR) spectroscopy. NMR Spectroscopy was carried out at 297 K on spectrometers from Bruker: Avance II and Avance II at frequencies of 400 MHz and 250 MHz for ^1H nuclei and 75 MHz for ^{13}C nuclei. Spectra were calibrated to the residual solvent peaks of dimethylsulfoxide-d_6 and chloroform-*d* at 2.50 ppm and 7.26 ppm,

respectively.[1] Data were processed with MestReNova (14.2) software and all chemical shifts δ are reported in parts per million (ppm) with coupling constant in Hz (multiplicity: s = singlet, d = doublet, dd = doublet of doublet, t = triplet, dt = doublet of triplet, ddd = doublet of doublet of doublet, sep = septet, m = multiplet, br = broad signal).

High-performance liquid chromatography (HPLC). Measurements were conducted using a Shimadzu HPLC LC-40D system, equipped with a Shim-pack GIST C18 column (150 mm length, 4.6 mm internal diameter, and 5 μm particle size), Each sample was dissolved in a mixture of ACN:water (1:1, v:v), supplemented with 0.1 % TFA, to obtain a concentration of ca. 10^{-5} M. The eluting solvent system consists of water (A) against ACN (B) as the following gradient: 0.0 min: 5 % B, 11.0 min: 38 % B, 24.5 min: 95 % B, 26.5 min: 95 % B, 27.0 min: 5 % B, 31.0 min: 5 % B. 10 to 30 μL were injected in the column, the flow rate was set at 1.0 mL/min and chromatograms were monitored at 274 nm. The data were acquired directly from the instrument in an ASCII format.

Ultraviolet-visible (UV-vis) spectroscopy. Solution phase and solid-state UV-vis spectra were recorded on a Shimadzu UV-1800 spectrophotometer. Quartz cuvettes (1 cm) from Thor Labs were used to measure the absorption of solutions, while solid polymer films were spin-coated onto quartz slides and placed in the cuvette holder. Absorption spectra were recorded from 200–400 nm, with 2 nm increments at a slow scan rate. The data were directly acquired from the instrument in comma-separated values (CSV) format.

Fourier-transform infrared (FTIR) Spectroscopy. FTIR measurements were carried out on an Agilent Cary 630 FTIR spectrometer equipped with a single bounce diamond ATR sampling accessory. The spectra were recorded in the spectral range of 4000 cm^{-1} to 650 cm^{-1} with a resolution of 4 cm^{-1} and 74 scans. The data were acquired from the MicroLab software in CSV format.

Gel-Permeation Chromatography (GPC). Experiments were performed on an Agilent 1260 Infinity II system equipped with a PolyPore Guard (ID = 7.5 mm, L = 50 mm) and two Polypore columns (ID = 7.5 mm, L = 300 mm, particle size = 5 µm). Signals were recorded by an interferometric refractometer detector (Agilent 1260 II series) and a multi-angle light scattering detector (Agilent 1260 II series, UV detector, 8 signals, 120 Hz). Measurements were conducted using THF as the eluent, at a temperature of 40 °C, and with a flow rate of 1.0 mL/min. Molecular weights were determined based on narrow molecular weight polystyrene calibration standards.

Photoreactors. Irradiations of solutions were carried out with Atlas Photonics Lumos 43 photoreactors equipped with LEDs of various wavelengths (ca. 5 nm half width). The power associated with the utilized LED is indicated in Table S1. The instrument features a power supply, a reactor, and sample holders compatible with standard NMR tubes, test tubes, and UV cuvettes (1 cm).

Wavelength (nm)	265	340
Power (mW)	22	33

Table S1: LED power of photoreactor.

Irradiation experiments of materials (blends, copolymers) were conducted using a custom-built photoreactor in the form of a cube, with one LEDs placed on the top surfaces (Fig. S29b). LEDs were purchased from Neumüller Elektronik (340 nm) and Boston Electronics (265 nm) and their power output are provided in Fig. S29a.

a)

Wavelength·[nm]	265	340
Power·[mW]	82	55

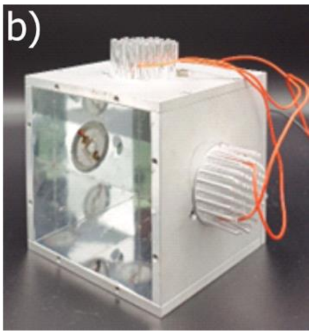

Figure S29. **a)** LED power of custom-built photoreactor. **b)** Photograph of the custom build photoreactor.

Photography. Photographs and videos were taken with Canon EOS R10 digital camera equipped with a Canon lens RF85mm F2 Macro IS STM. The video was edited with the free and open source video editor Shotcut.

Differential Scanning Calorimetry (DSC). DSC experiments were carried out under nitrogen atmosphere on a Netzsch 204 F1 Phoenix DSC instrument operating at a heating/cooling rate of 5 °C/min in the range of 25 to 300 °C. The maximum temperature varied depending on the experiments, ranging between 180 °C and 300 °C. A 5-min isotherm was applied at 25 °C and at the maximum measurement temperature for all experiments. Measurements were performed in standard aluminum pans using 5–15 mg of sample.

Thermogravimetric Analysis (TGA) experiments were carried out under nitrogen atmosphere on a Netzsch STA 449 F5 thermo-gravimetric analyzer. Samples were heated from 25 to 500 °C at a rate of 5 °C /min for **QM**, **QD** components, and for polymer blends. Copolymers were

measured at a rate of 10 °C/min. All measurements were conducted under a nitrogen atmosphere.

Atomic Force Microscopy (AFM). Indentation measurements were performed with a MFP3D (Asylum Research, an Oxford Instruments company) in air at ca. 21 °C with a biosphere B50-FM cantilever (high-density diamond-like carbon spherical tip, radius: (50 ± 5) nm, NanoAndMore). For calibration, the inverse optical lever sensitivity (invOLS) was determined by indentation into a freshly cleaved mica sample (muscovite, diameter 10-12 mm, Plano), averaging five invOLS values at the beginning and end of any experimental set, respectively. The force constant value indicated by the manufacturer was taken (pre-calibrated cantilever). Usually a deflection trigger value (proportional to the indentation force) of 0.4 V (ca. 100 nN) was used and the dwell time was set to 0. Approach and retraction velocities were usually kept constant at 1.0 µm/s with a sampling rate of 2 kHz. Force curves were obtained by contacting the underlying surface in a grid-like fashion (force map), i.e., force maps with 10 by 10 points on an area of 10 µm by 10 µm were taken, resulting in 100 force curves per force map. Usually, five to six force maps with the same parameters were taken at different locations on the sample. Thus, the obtained values are representative for the respective sample.

The evaluation was done using the AFM software to determine the elastic moduli (elastic panel in the AFM software) of the respective sample. The baselines of the force-distance curves were corrected for drift. For the elastic moduli determination, the Hertz model [1] was used, with the following setting for the cantilever tip: a sphere of a radius of 50 nm, an elastic modulus of 865 GPa and a Poisson ratio of 0.2 (software settings for synthetic diamond, see also Dang et al.[2]). Furthermore, a Poisson ratio of 0.33[2] was assumed for the sample. A maximum indentation portion of 10 nm was fitted. Krieg et al. provide an overview of the respective functions and rules of thumb for evaluation process.[3] The distribution of values was plotted as

histograms. A Gaussian was fitted to each histogram peak to obtain the mean value as the maximum of the Gaussian and the error as the width of the Gaussian.

Creep experiment.

Sample preparation: A 1 % w/v solution of **PQM** in chloroform was prepared and degassed using ten freeze–pump–thaw cycles. In an inert atmosphere glovebox, 20 µL of this solution was solvent-cast onto a 1 cm^2 area of a glass slide (1 cm × 2 cm). The coated slides were annealed under vacuum overnight to remove residual solvent. To prepare lap joints, two coated slides were aligned with their coated areas overlapping (1 cm^2 contact area) and fixed using clamps. The assembled joints were annealed under inert conditions at 80 °C for 1 h to promote bonding between the two films. After cooling, the samples were left to rest for 4 h before testing.

Creep test procedure: Samples were loaded with either 600 g or 1200 g weights, corresponding to forces of approximately 5.9 N and 11.8 N, respectively. A Logitech C270 webcam was used to record the displacement of the bonded joint at a frame rate of 1 frame per second.

Image analysis and displacement extraction: To track displacement, contrast marks were applied to the sample surface. From each frame, a rectangular region of interest (ROI) was selected around the mark using ImageJ. For each frame, the grayscale intensity profile across the ROI was calculated by averaging pixel values along the vertical direction, producing a one-dimensional intensity trace along the *x*-axis for each time point. These intensity profiles were exported and analyzed to extract the position of the moving edge with subpixel accuracy. For each profile, the region around the steepest intensity gradient was identified. Within this region (±4 pixels), a Gaussian function with offset was fitted to the original intensity data. The peak

position of the Gaussian was taken as the refined x-position of the marker. This process yielded time-resolved displacement data with subpixel resolution.

2.2 Methods and Procedures

2.2.1 General procedure for molecular blends in PMMA

The molecular component (**QM**, **QD**) was weighed into a glass vial and dissolved in chloroform under stirring at 1000 rpm for 10 min. PMMA powder was then added to the solution and stirred until completely dissolved (~ 1 h). The resulting homogeneous solution was solvent-cast into aluminum trays (Ø = 57 mm) and dried under atmospheric conditions on a flat surface for 24 h. Subsequently, the polymer films were annealed for at least 1 h at 150 °C under reduced pressure (200 mbar) and stored in a desiccator.

2.2.2 General procedure for molecular blends in PEHMA

PEHMA and the molecular component (**QM** or **QD**) were weighed into a glass vial to prepare a chloroform solution containing 1 wt% **QM** or **QD** relative to **PEHMA**. Chloroform has been pre-filtered over neutral aluminum oxide. The solution was degassed using 5–10 freeze–pump–thaw cycles, then 200 µL were drop-cast onto microscope slides inside a glove box under inert atmosphere. For creep experiments, 20 µL were cast onto a defined area of 1 cm^2. After solvent evaporation, the films were annealed overnight at room temperature (~25 °C) under high vacuum ($p < 1$ mbar).

2.2.3 Film preparation of copolymers

PQM was dissolved at 1 wt/vol % in chloroform previously filtered over neutral aluminum oxide. The solution was degassed by 5–10 freeze–pump–thaw cycles, then 200 µL were drop-cast onto a microscope slide inside a glove box under inert atmosphere. After solvent evaporation, the film was annealed overnight at ~25 °C under vacuum ($p < 1$ mbar).

2.2.4 General procedure for photoreactions in solution

The adduct was solubilized in pre-dried and distilled acetonitrile at a $c = 4\text{-}6 \cdot 10^{-5}$ mol/L. The solution was degassed through three freeze-pump-thaw cycles, and subsequently placed inside a glovebox. A 3 mL aliquot of this oxygen-free solution was carefully transferred into a quartz cuvette, which was then sealed with a PTFE stopper. To ensure an airtight seal, parafilm was wrapped around the stopper before the cuvette was removed from the glovebox.

2.2.5 Photocycloaddition in solution

Cycloaddition reactions were carried out using the dimer, reverted through DSC experiments, at a concentration range of $4\text{-}6 \cdot 10^{-5}$ mol/L. The cuvettes were positioned in the photoreactor using a sample holder. Irradiations were conducted at 340 nm, and UV-vis spectra were recorded at intervals ranging from 10 seconds to 2 min until a plateau was reached, typically within 5 to 20 min. It is worth noting that due to the experimental setup, real-time measurements could not be performed, and the irradiation kinetics needed to be momentarily halted for measurement purposes. Furthermore, to mitigate the influence of ongoing irradiation during UV-vis measurement, absorptions were recorded at the quinolinone's maximum absorption wavelength, specifically $\lambda = 328$ nm. Full spectral measurements were exclusively conducted for the reference spectra (sample at $t = 0$ min before irradiation), allowing for the subsequent conversion calculations. The conversion c_t at time t was calculated by the following equation:

$$p = \left(1 - \frac{A_{,\lambda=328\ \mathrm{nm}}}{A_{t=0,\lambda=328\ \mathrm{nm}}}\right) \cdot 100\ \%$$

where $A_{\lambda=328\ \mathrm{nm}}$ and $A_{t=0,\lambda=328\ \mathrm{nm}}$ are the absorptions at time t and $t = 0$ min, respectively. The absorptions of quinolinone were measured at 328 nm.

2.2.6 Photocycloaddition in solid state

Cast films were prepared by dissolving 500 mg of PMMA and 1 mg of QM in chloroform. The solvent was allowed to evaporate, forming a film approximately 130 µm thick, cast in an alumina dish. The resulting film was cut into pieces measuring approximately 1 cm × 2 cm and placed in a quartz cuvette sealed with PTFE. All steps up to this point were carried out inside a glove box. The films were irradiated at 340 nm for the cycloaddition reaction and at 265 nm for the cycloreversion reaction. Conversion was monitored following the procedure described in Section 2.2.4.

2.2.7 Cycling procedure – photocycloaddition and thermal cleavage

A stock solution (1 mg/mL of **QD**) was prepared, and 35 µL was mixed with 3 mL of acetonitrile. A UV measurement was performed to determine the initial concentration. The solvent was then evaporated under high-pressure vacuum, and the cuvette was placed in a Schlenk tube and heated at approximately 210 °C for 1 h. The cuvette containing the heated product was subsequently, transferred to a glovebox, where 3 mL of degassed acetonitrile (prepared through three cycles of freeze-pump-thaw) was added, and a UV-vis spectrum was recorded. The solution was irradiated at 340 nm for 12 min in a photoreactor, followed by another UV-vis measurement. The solvent was then evaporated under high-pressure vacuum, and a new heating cycle was applied. This process was repeated for a total of three cycles. Conversions for the reversion reactions were calculated using the equation discussed in Section 2.2.4.

2.2.8 Thermally-induced Cycloreversion Kinetics (Molecular State)

A saturated stock solution of **QD** (500 µL) in $CDCl_3$ was transferred to an NMR tube, and an additional 100 µL of $CDCl_3$ was added to prevent crystallization. The initial concentrations of

QD and **QM** were quantified using ^{1}H NMR spectroscopy. Subsequently, $CDCl_3$ was evaporated under vacuum (<0.001 bar). The sample was then placed in an oil bath with temperature control maintained by thermostat equipped with a PT100 sensor, ensuring stable temperatures of 180 °C, 190 °C, 200 °C, and 210 °C. The reaction was conducted for various time intervals ranging from 30 seconds to 1 h, and the conversion was monitored using ^{1}H NMR spectroscopy. For analysis, the sample was dissolved in 600 µL of $CDCl_3$, which was again removed before resuming reaction monitoring in the solid state. The reaction was carried out until full conversion was achieved.

2.2.9 Procedure to conduct the kinetics through the use of Arrhenius Plots

^{1}H NMR spectroscopy was employed to determine the **QD** and **QM** ratio and to track the reaction kinetics of the conversion over time. The concentration ratio of **QD** to **QM** ($c_{\mathrm{QD}}/c_{\mathrm{QM}}$) was calculated by comparing the integrated peak area of the N-CH_3 group of **QD** (A_{QD}, integrated between 3.533–3.229 ppm) with that of the vinyl and aromatic protons of **QM** (A_{QM}, integrated between 7.743–7.368 ppm). The ratio was then calculated as:

$$\frac{c_{\mathrm{QD}}}{c_{\mathrm{QM}}} = \frac{A_{\mathrm{QD}}/3}{A_{\mathrm{QM}}/2}.$$

For the homolytic cleavage reaction **QD**→2**QM**, the concentration of **QD** at time $t = 0$ can be expressed in terms of:

$$c_{\mathrm{QD},t=0} = c_{\mathrm{QD}} + \frac{c_{\mathrm{QM}}}{2},$$

where c_{QD} is the concentration of **QD** at a time (t). The conversion (p) is expressed by:

$$p = \frac{c_{\mathrm{QD}}}{c_{\mathrm{QD,t=0}}} = \frac{1}{1 + \frac{c_{\mathrm{QM}}}{2c_{\mathrm{QD}}}}$$

The reaction rate constant (k) is determined from the linear regression of $\ln(c_{\mathrm{QD}}/c_{\mathrm{QD,0}})$ versus time (t):

$$\ln(c_{\mathrm{QD}}/c_{\mathrm{QD},0}) = -k\, t.$$

The activation energy (E_{a}) is obtained from the Arrhenius equation, with the attempt frequency A:

$$\ln(k) - \ln(A) = -\frac{E_{\mathrm{a}}}{RT}.$$

A linear regression of the Arrhenius plot ($\ln(k)$ versus $1/T$) yields the slope $-\frac{E_{\mathrm{a}}}{R}$ and a y intercept of $\ln(A)$.

2.2.10 Gel point determination

Photocycloaddition reaction was performed according to the procedure described in Section 2.2.5, using irradiation intervals ranging from 0 to 10 min. The sol fraction of the irradiated films was extracted with 3 mL of chloroform, and the absorption spectra were recorded using UV-vis spectroscopy. In order to correct for the concentration of the sample, the absorption spectra are normalized by the absorption for the isosbestic point (274 nm). The gel point is defined as the irradiation time point at which the **PQM** concentration in the solvent phase exhibits a sudden, stepwise decrease.

2.3 Synthetic Procedures and Analytical Data

2.3.1 Synthesis of 7-methoxy-1-methylquinolin-2-one (QM).

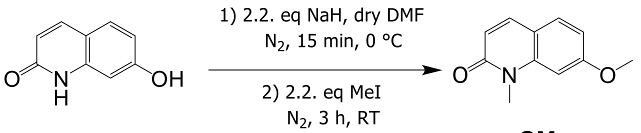

QM

7-methoxy-1-methylquinolin-2-one (**QM**) was synthesized according to previously reported procedures.[4] Sodium hydride (60 % suspension in paraffin wax) (1.64 g, 40.96 mmol, 2.2 eq.) was put in a flame-dried two-necked 100 mL round-bottom flask and purged with nitrogen. The flask was then cooled down with an ice-bath before injecting dry DMF (30 mL). Then, 2,7-dihydroquinoline (iminol form of 7-hydroxyquinolin-2(1H)-one) (3.00 g, 18.60 mmol, 1.0 eq.) was slowly added to the suspension, stirred for 15 min at 0 °C, and then iodomethane (2.55 mL, 5.81 g, 40.96 mmol, 2.2 eq.) was added dropwise. The reaction mixture was allowed to warm to room temperature (ca. 22 °C) and stirred for 3 h. Upon completion of the reaction (monitored by TLC using EtOAc:CyH (9:1) as eluent), the solution was quenched with water (ca. 20 mL), washed with EtOAc (ca. 40 mL) and transferred into a separating funnel. The aqueous phase was partitioned and extracted three times with EtOAc (ca. 40 mL). The organic layers were combined and washed with 100 mL of water (one time) and 100 mL of brine (three times). The organic phase was then dried over $MgSO_4$, filtered, evaporated and fully dried under reduced pressure. The crude product was purified by flash column chromatography, using EtOAc:CyH ((9:1), (v:v)) as eluent, to afford **QM** (2.15 g, 11.35 mmol, 61 %) as a white solid. ^{1}H-NMR (300 MHz, $CDCl_3$) δ (ppm): 7.59-7.56 (d, 1H, vinyl -C*H*), 7.45-7.43 (d, 1H, Ar*H*), 6.82–6.79 (dd, 1H, Ar*H*), 6.77 (m, 1H, Ar*H*), 6.55-6.52 (d, 1H, vinyl -C*H*), 3.91 (s, 3H, -OCH_3), 3.67 (s, 3H, -NCH_3). ^{13}C-NMR (75.4 MHz, $CDCl_3$) δ (ppm): 162.89, 161.93, 141.87, 138.85, 130.23, 118.67, 115.05, 109.72, 98.80, 55.73, 29.59. HRMS, ESI, (m/z) [M] calc. for $C_{11}H_{12}NO_2$: 190.08; found: 190.08.

2.3.2 Synthesis of QD$_{aHH}$

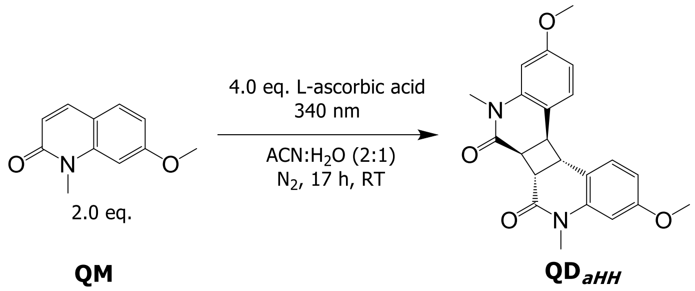

QM (1 g, 5.3 mmol, 2.0 eq.) was dissolved in acetonitrile (17 mL) inside a Schlenk tube under stirring and a nitrogen atmosphere. After heating slightly to obtain a clear solution, the reaction mixture was allowed to warm to room temperature (ca. 22 °C) and L-ascorbic acid (1.83 g, 10.6 mmol, 4.0 eq.) was added and dissolved in water (8 mL). The solution was then irradiated using a 340 nm LED (55 mW) for 17 h, then cooled in an ice bath to fully precipitate the desired **QD$_{aHH}$**. The white precipitate formed was filtrated, washed with water (20 mL) and dried under vacuum, to afford **QD$_{aHH}$** (226.0 mg, 0.60 mmol, 22.6 %) as a white solid. ^{1}H-NMR (300 MHz, $CDCl_3$) δ (ppm): 6.84-6.82 (d, 2H, Ar*H*), 6.59-6.59 (d, 2H, Ar*H*), 6.54-6.51 (dd, 2H, Ar*H*), 3.82 (s, 6H, -OC*H*3), 3.71-3.70 (m, 4H, cyclobutane-C*H*), 3.43 (s, 6H, -NC*H*3). ^{13}C NMR (75 MHz, $CDCl_3$) δ (ppm): 169.45, 159.88, 140.87, 128.55, 116.12, 106.87, 102.88, 55.57, 43.92, 43.54, 29.83. HRMS, ESI, (m/z) $[M—Na]^+$ calc. for $C_{22}H_{22}N_2O_4$: 401.1472; found: 401.1474.

2.3.3 Synthesis of 7-hydroxy-1-methylquinolin-2(1H)-one (1)

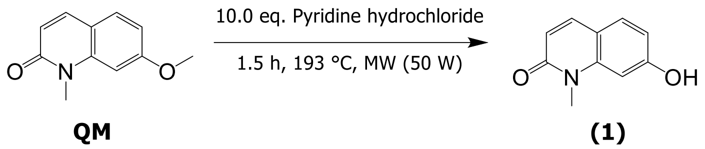

Compound (**1**) was synthesized according to previously reported procedures.[6] **QM** (1.0 g, 5.28 mmol, 1.0 eq.) and pyridine hydrochloride (6.1 g, 52.8 mmol, 10.0 eq.) were combined and well mixed in a 35 mL microwave vial. The reaction was performed under microwave

irradiation (193 °C, 50 W) under high stirring for 1.5 h. The resulting brown solid was then poured into iced water (100 mL) under stirring. The white suspension was isolated by filtration and washed with additional water (50 mL). The remaining solid was dried under high vacuum and purified by flash column chromatography, using EtOAc as eluent to afford **1** (415.9 mg, 2.38 mmol, 45 %) as a white powder. ^{1}H NMR (300 MHz, DMSO-d_6) δ (ppm): 10.25 (s, 1H, -O*H*), 7.76-7.73 (d, 1H, vinyl -C*H*-), 7.53-7.50 (d, 1H, Ar*H*), 6.80–6.70 (m, 2H, Ar*H*), 6.36-6.33 (d, 1H, vinyl -C*H*-), 3.52 (s, 3H, -NCH_3). ^{13}C NMR (75.4 MHz, DMSO-d_6) δ (ppm): 161.49, 160.16, 141.69, 139.11, 130.31, 116.62, 113.17, 111.08, 100.00, 28.86.

2.3.4 Synthesis of 7-(2-hydroxyethoxy)-1-methylquinolin-2(1H)-one (2)

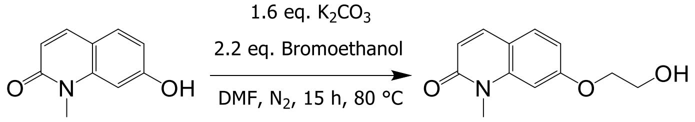

Compound (**2**) was synthesized according to previously reported procedures.[6] Compound (**1**) (276.0 mg, 1.58 mmol, 1.0 eq.) and K_2CO_3 (350.0 mg, 2.53 mmol, 1.6 eq.) were dissolved in dry DMF (8 mL) in a flame-dried Schlenk flask (25 mL), under a nitrogen atmosphere. The reaction mixture was heated to 80 °C and stirred for 15 min before adding 2-bromoethanol (440.7 mg, 0.25 mL, 3.53 mmol, 2.2 eq.) dropwise. The mixture was then further stirred at 80 °C for an additional 15 h. Upon completion of the reaction (monitored by TLC using EtOAc as eluent), the solvent was evaporated under reduced pressure. The residue was dissolved in dichloromethane, adsorbed onto ca. 100 mg of SiO_2, and purified by silica gel flash column chromatography. First, EtOAc was used to remove unreacted starting material, followed by elution with EtOAc:MeOH (97:3) to afford **2** (226.6 mg, 1.03 mmol, 66 %) as a pale orange solid. ^{1}H NMR (300 MHz, DMSO-d_6) δ (ppm): 7.82-7.79 (d, 1H, vinyl-C*H*), 7.64-7.61 (d, 1H, Ar*H*), 6.94-6.88 (m, 2H, Ar*H*), 6.43-6.40 (d, 1H, vinyl-C*H*), 4.95-4.91 (t, 1H, -O*H*), 4.16-4.13 (t, 2H, O-$CH_2C\mathit{H_2}OH$), 3.79-3.74 (m, 2H, O-$C\mathit{H_2}CH_2OH$), 3.59 (s, 3H, -NCH_3). ^{13}C NMR

(75.4 MHz, DMSO-d_6) δ (ppm): 161.43, 160.95, 141.45, 138.93, 130.14, 117.59, 114.12, 110.17, 99.31, 70.00, 59.50, 29.01. HRMS, ESI, (m/z) $[M—Na]^+$ calc. for $C_{12}H_{13}NO_3Na$: 242.08; found: 242.0788.

2.3.5 Synthesis of quinolinone methacrylate (QMMA)

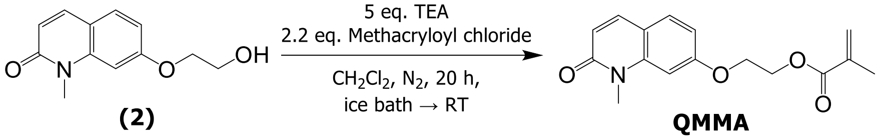

QMMA was synthesized according to previously reported procedures.[6] Compound (**2**) (544.30 mg, 2.48 mmol, 1.0 eq.) was added to a dry 250 mL round bottom flask and consecutively subjugated to three nitrogen/vacuum cycles. Dichloromethane (35 mL) and triethylamine (1.73 mL, 1.26 mg, 12.41 mmol, 5 eq.) were added to solubilize compound **2** and the reaction was stirred in an ice bath for 15 min. Methacryloyl chloride (0.48 mL, 0,52 mg, 4.97 mmol, 2 eq.) was then added dropwise, and the reaction was allowed to warm to room temperature (ca. 22 °C) and proceed for 20 h. The reaction mixture was quenched with water (50 mL) and the organic layer was subsequently washed two times with brine (50 mL). The aqueous phases were collected and extracted two times with dichloromethane (50 mL). The organic layers were collected, evaporated under reduced pressure, and purified by silica gel flash column chromatography using EtOAc:CyH (95:5), (v/v)) as eluent. The product was then dried under high vacuum to afford **QMMA** (335 mg, 1.17 mmol, 61 %) as pale brown powder. ^1H NMR (400 MHz, $CDCl_3$) δ 7.61-7.59 (d, 1H, vinyl-C*H*), 7.49-7.46 (d, 1H, Ar*H*), 6.85-6.83 (m, 2H, Ar*H*), 6.58-6.56 (d, 1H, vinyl-C*H*), 6.16-6.15 (dq, 1H, acrylate-CH_{cis}), 5.61-5.60 (m, 1H, acrylate-CH_{trans}), 4.56–4.54 (m, 2H, -OCH_2), 4.35–4.32 (m, 2H, -OCH_2), 3.67 (s, 3H, -NCH_3), 1.96-1.95 (dd, 3H, -CH_3). ^{13}C NMR (101 MHz, $CDCl_3$) δ (ppm): 167.44, 160.85, 141.82, 138.82, 136.02, 130.30, 126.45, 118.99, 115.39, 109.95, 99.81, 66.42, 62.93,.29.65, 18.45.

2.3.6 Synthesis of PEHMA-ran-PQMMA

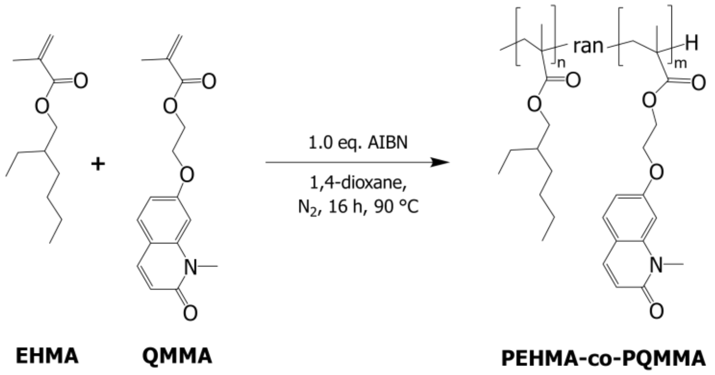

QMMA (33 mg, 0.12 mmol, 4.0 eq.), 2-ethylhexyl methacrylate (**EHMA**) (234 µL, 0.206 g, 1.04 mmol, 36.0 eq.) and AIBN (4,7 mg, 0.029 mmol, 1.0 eq.) were dissolved in 1,4-dioxane (0.75 mL) in a Schlenk tube. The solution was purged with nitrogen for 30 min, after which the Schlenk tube was sealed and heated to 90 °C for 16 h. The reaction mixture was then precipitated with 10 mL of cold aqueous methanol mixture (v:v 10 % H_2O). The resulting polymer was isolated by filtration, washed two times with 2 mL of methanol and dried under reduced pressure to afford PEHMA-co-PQMMA (0.210 g, 85 %) as a colorless solid. ^{1}H NMR (400 MHz, CDCl3) δ (ppm): 7.61-7.59 (br, 1H, QMMA: vinyl-C*H*), 7.47 (br, QMMA: 1H, Ar*H*), 6.85 (br, QMMA: 2H, Ar*H*), 6.57-6.55 (br, QMMA: 1H, vinyl-C*H*), 4.33-4.26 (br, QMMA: 4H, O-C*H*2-C*H*2-O), 3.83 (br, EHMA: 2H, C*H*2-CH), 1.90-1.80 (br, 2H, EHMA: C*H*2-CH-(CH2)2), 1.53 (br, 2H, C- EHMA: C*H*2-C), 1.29 (br, 12H, EHMA: C*H*3-C, C*H*3-CH2-C, C-C*H*2-C*H*2-C*H*2-CH3), 1.03-0.88 (br, 6H, EHMA: CH2-C*H*3, CH2-C*H*3). GPC: M_n = 15.2 10^3 g/mol, M_w = 26.6 10^3 g/mol, Đ = 1.75. Average degree of functionalization f = 5.7.

2.3.7 Synthesis of PEHMA

PEHMA was synthesized according to the procedure described in 2.3.6 using 2-ethylhexyl methacrylate (**EHMA**) as sole monomer. ^{1}H NMR (400 MHz, CDCl3) δ (ppm): 3.83 (br, EHMA: 2H, C*H*2-CH), 3.70 (br, 3H, QMMA: N-C*H*3), 1.90-1.81 (br, 2H, EHMA: C*H*2-CH-

(CH2)2), 1.56 (br, 2H, C- EHMA: C*H*2-C), 1.30 (br, 12H, EHMA: C*H*3-C, C*H*3-CH2-C, C-C*H*2-C*H*2-C*H*2-CH3), 1.03-0.90 (br, 6H, EHMA: CH2-C*H*3, CH2-C*H*3). GPC: M_n = 8.63 10^3 g/mol, M_w = 14.3 10^3 g/mol, Đ = 1.66.

Supporting Molecular Characterization

^{1}H-NMR spectrum of **QM** in $CDCl_3$.

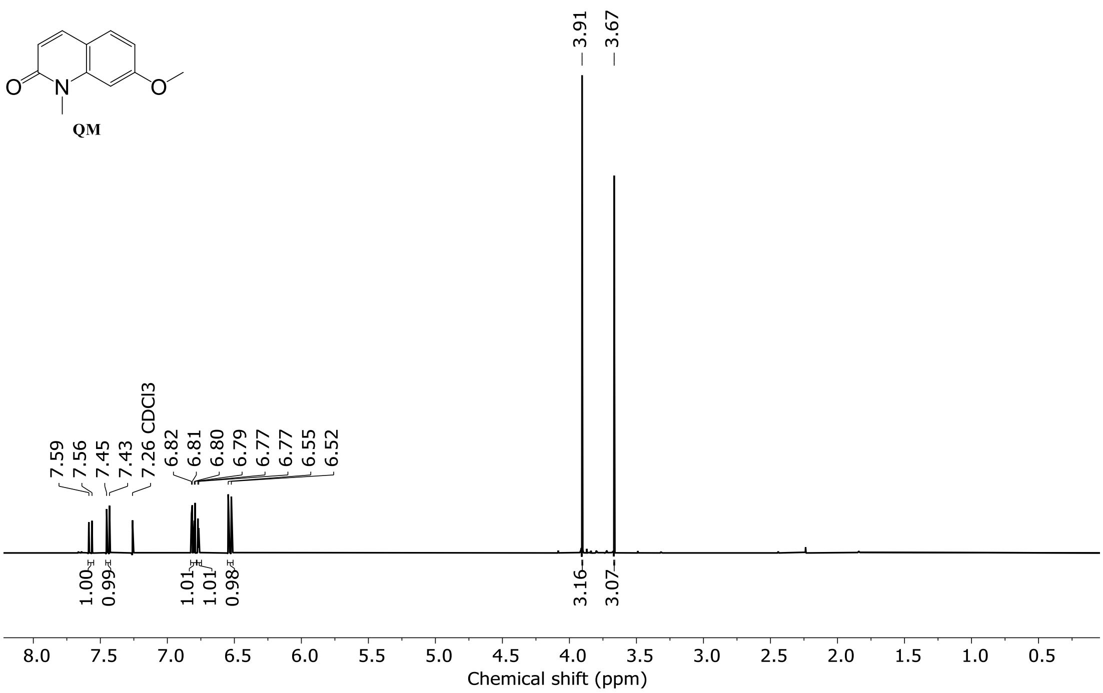

^{13}C-NMR spectrum of **QM** in $CDCl_3$.

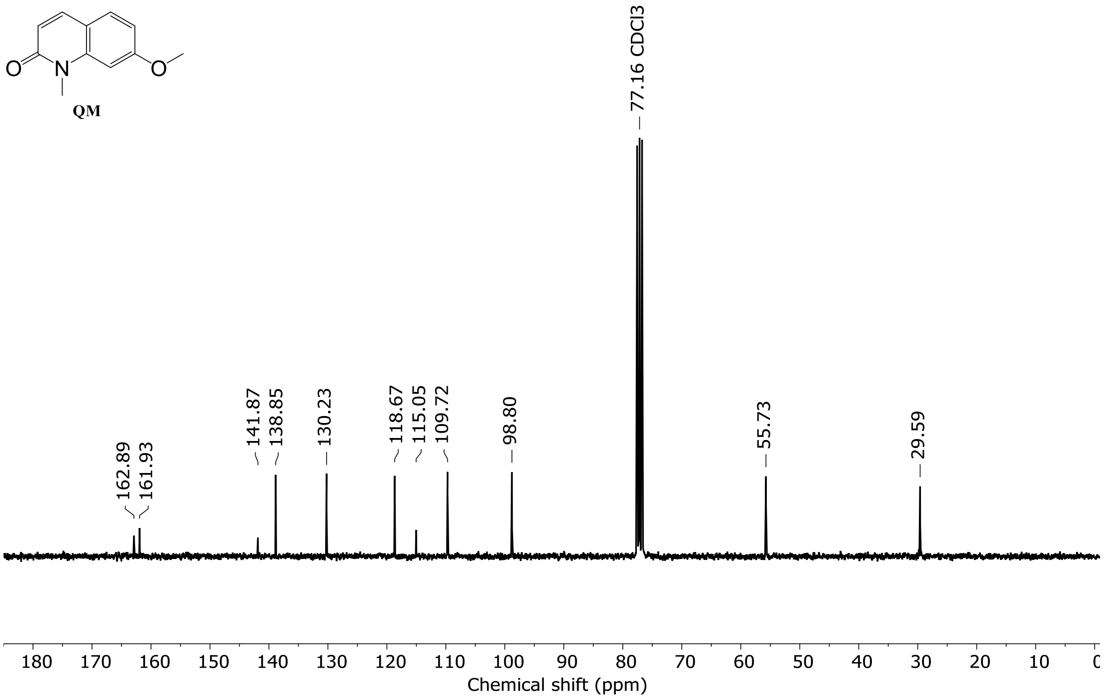

ESI spectrum of **QM**.

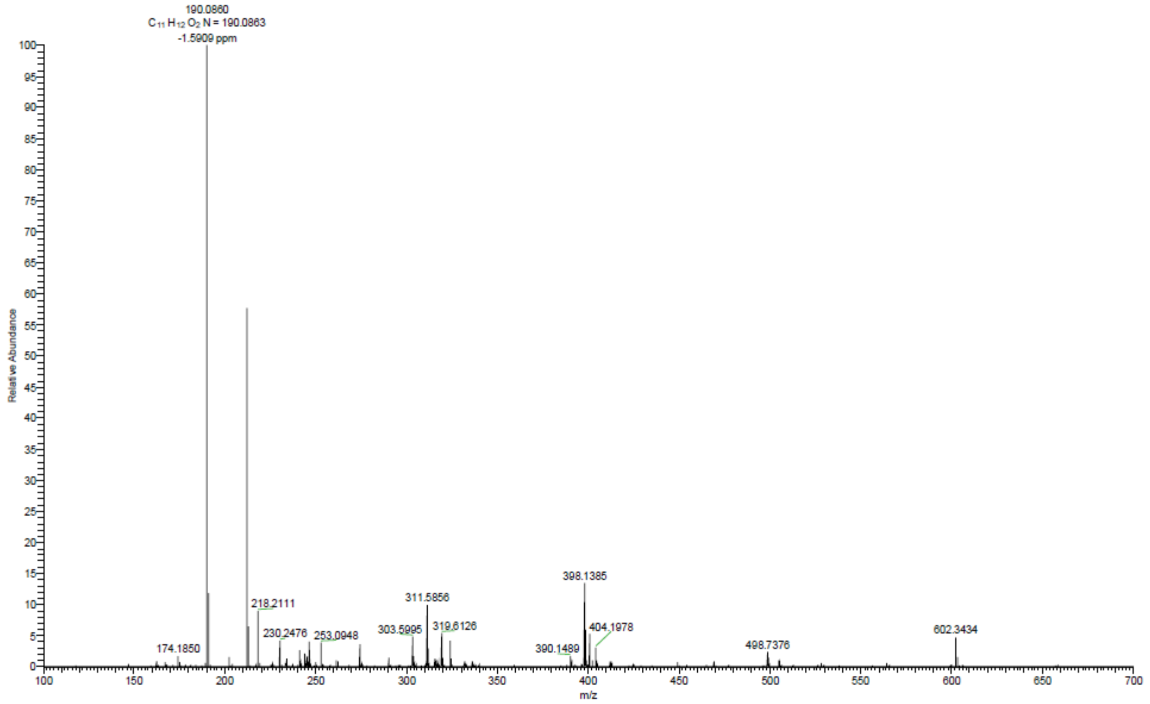

^{1}H-NMR spectrum of **QD*aHH*** in $CDCl_3$.

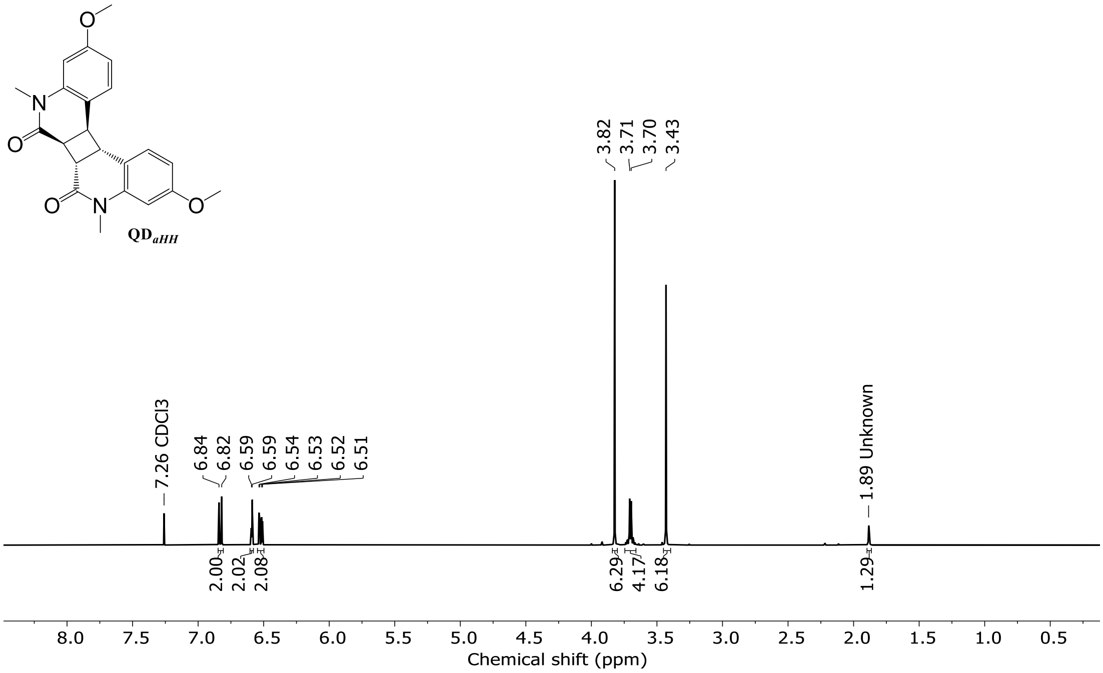

^{13}C-NMR spectrum of **QD*aHH*** in $CDCl_3$.

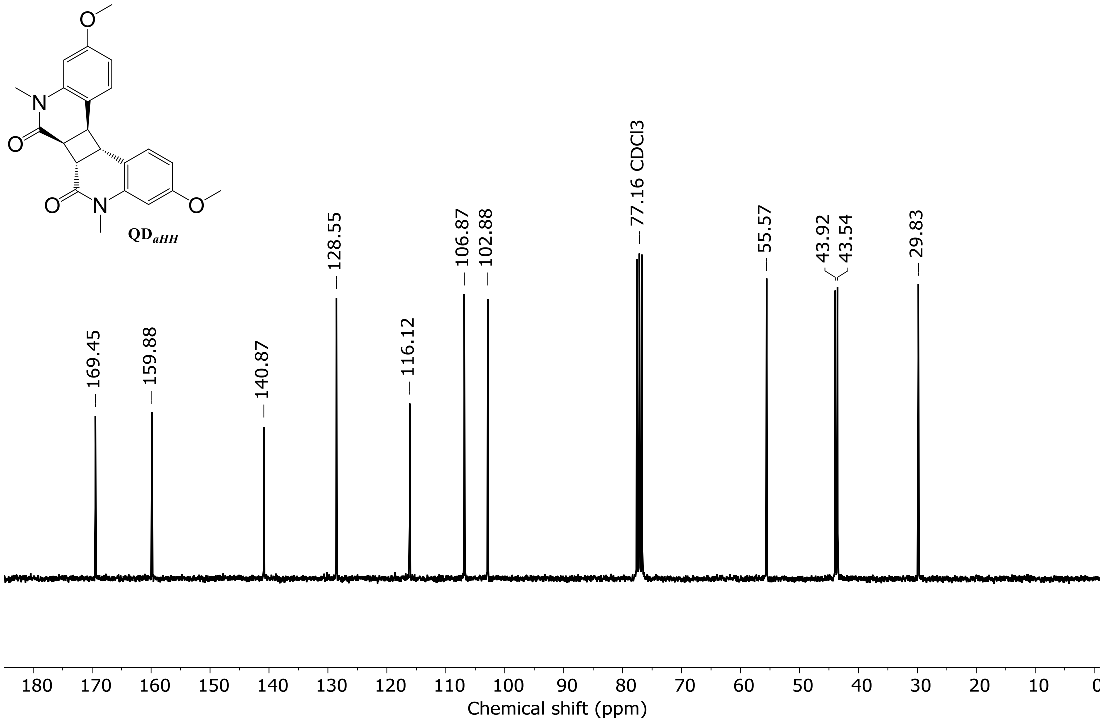

ESI spectrum of **QD**$_{aHH}$.

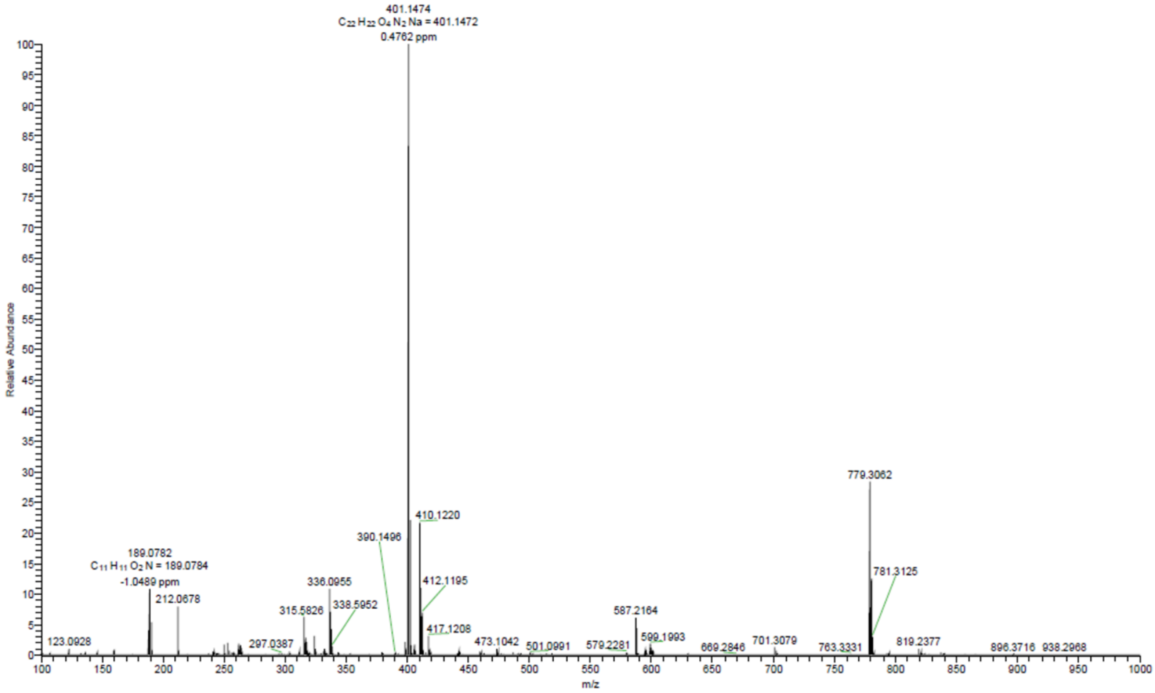

^{1}H-NMR spectrum of **(1)** in DMSO-d_6.

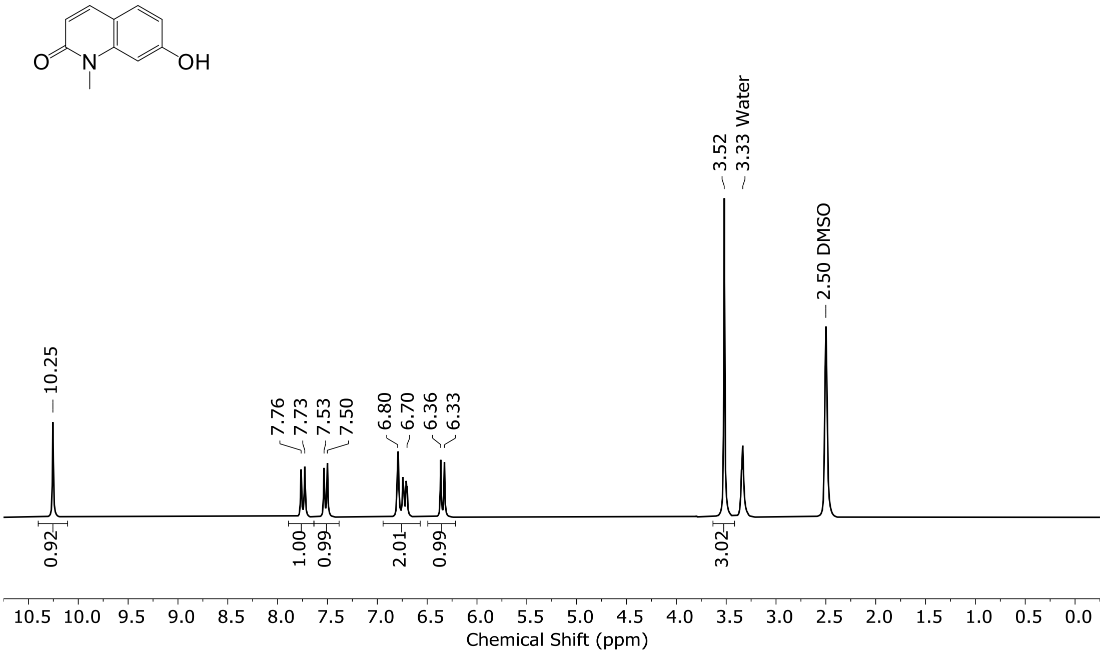

^{13}C-NMR spectrum of **(1)** in DMSO-d_6.

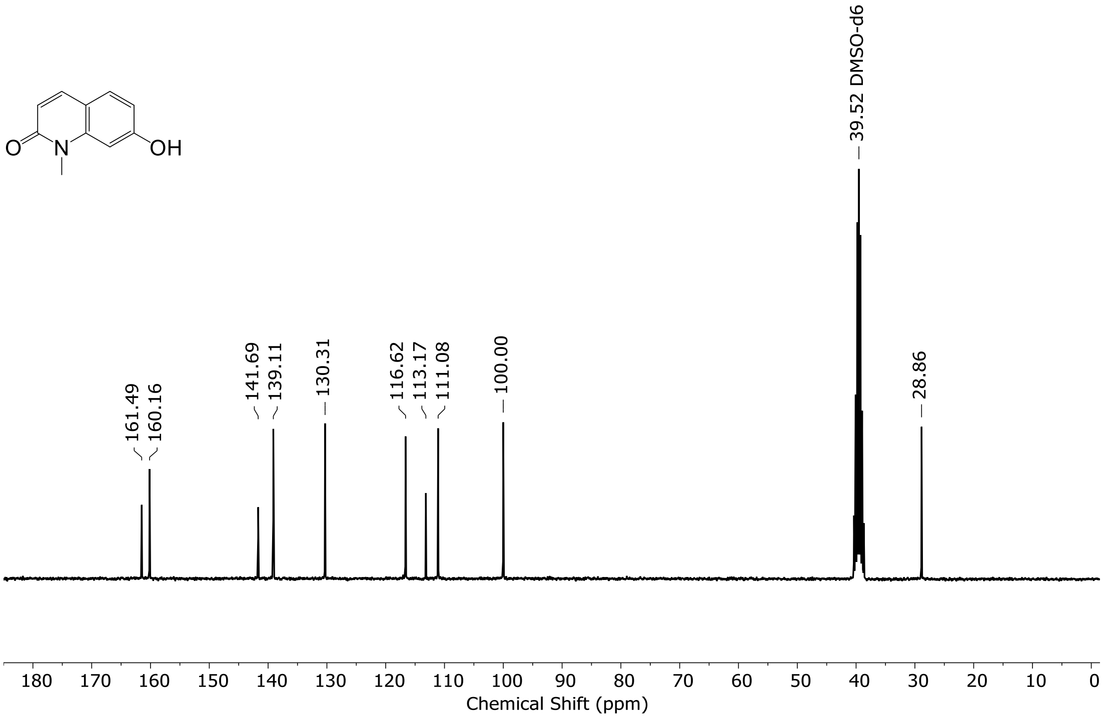

^{1}H-NMR spectrum of **(2)** in DMSO-d_6.

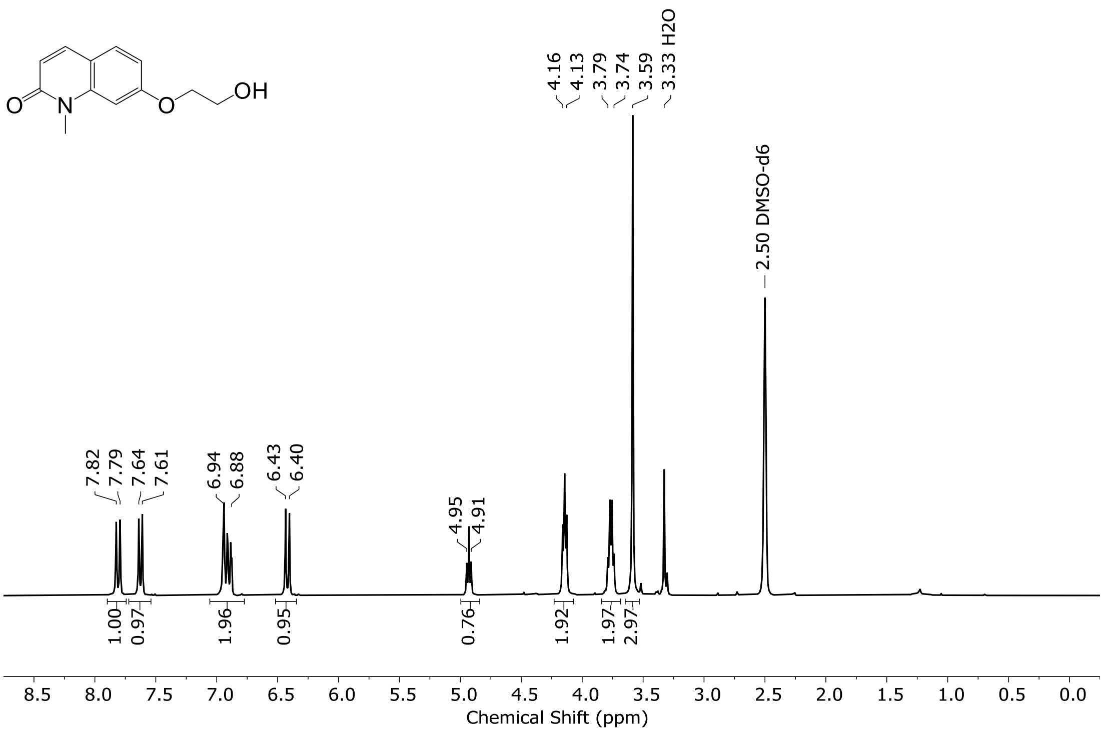

^{13}C-NMR spectrum of (**2**) in DMSO-d_6.

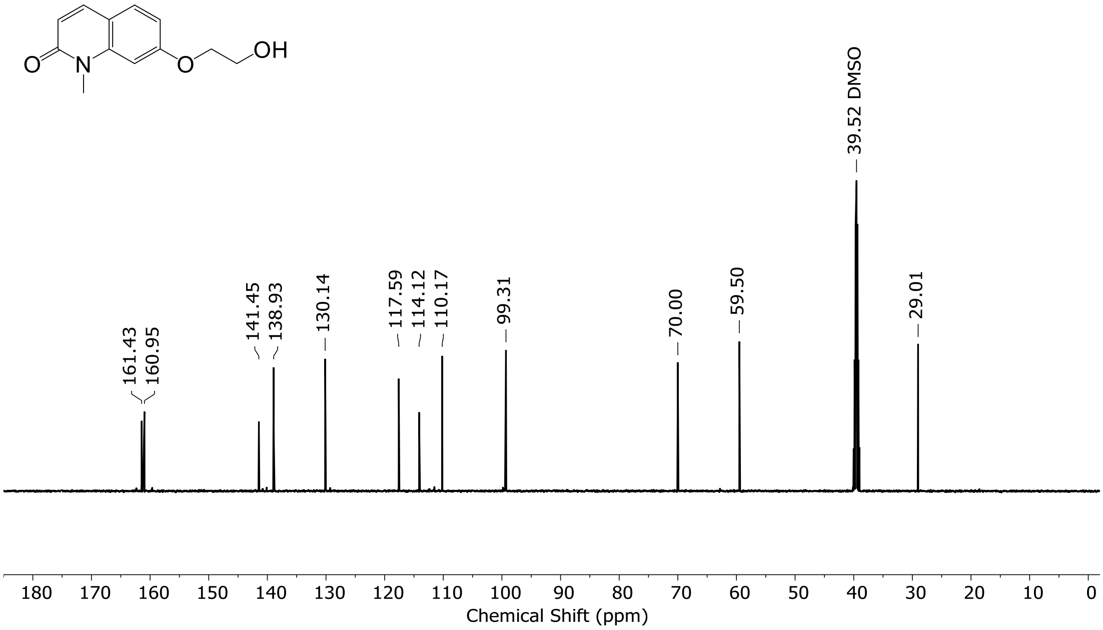

ESI spectrum of **(2)**.

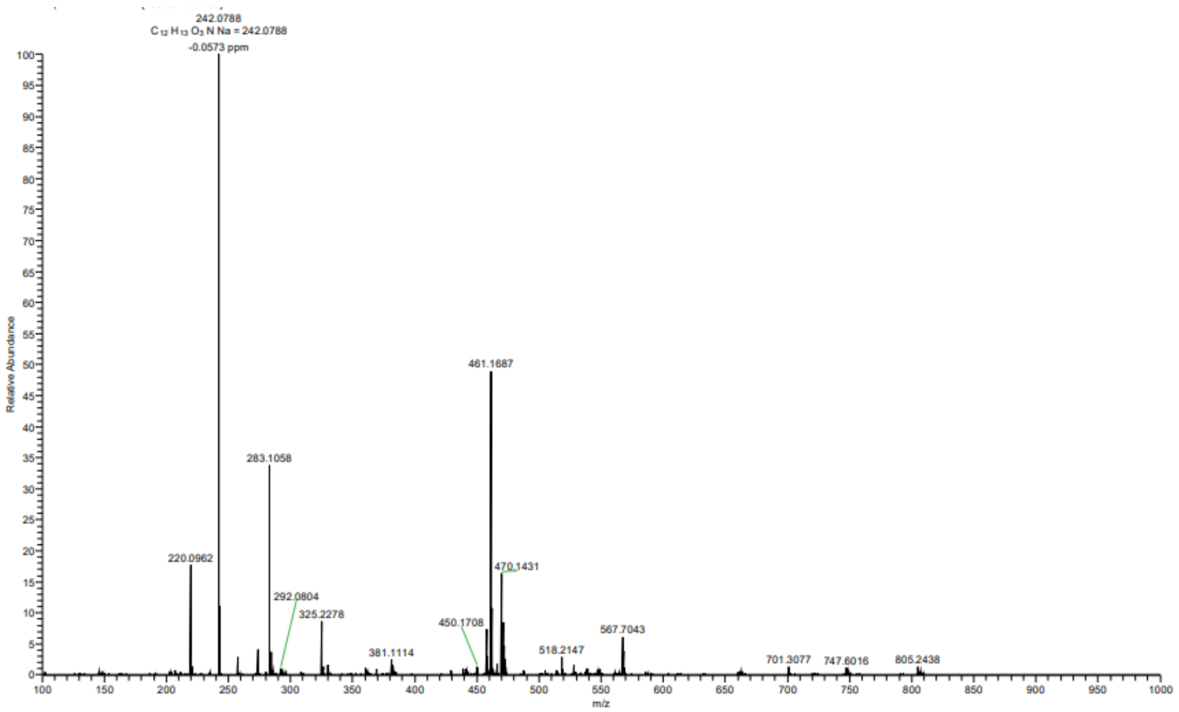

^{1}H-NMR spectrum of **QMMA** in $CDCl_3$.

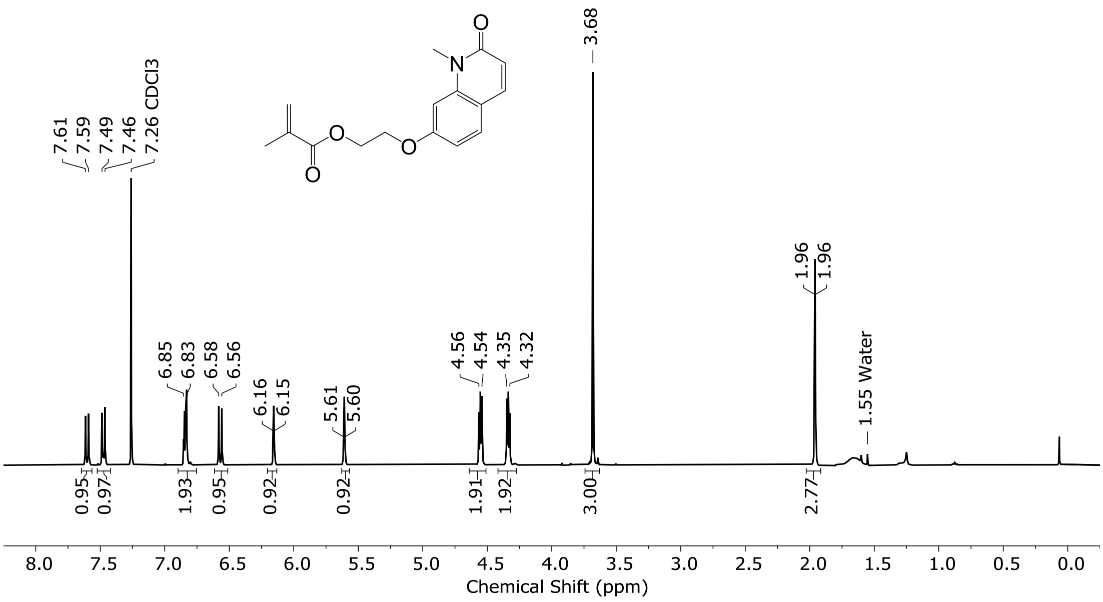

^{13}C-NMR spectrum of **QMMA** in $CDCl_3$.

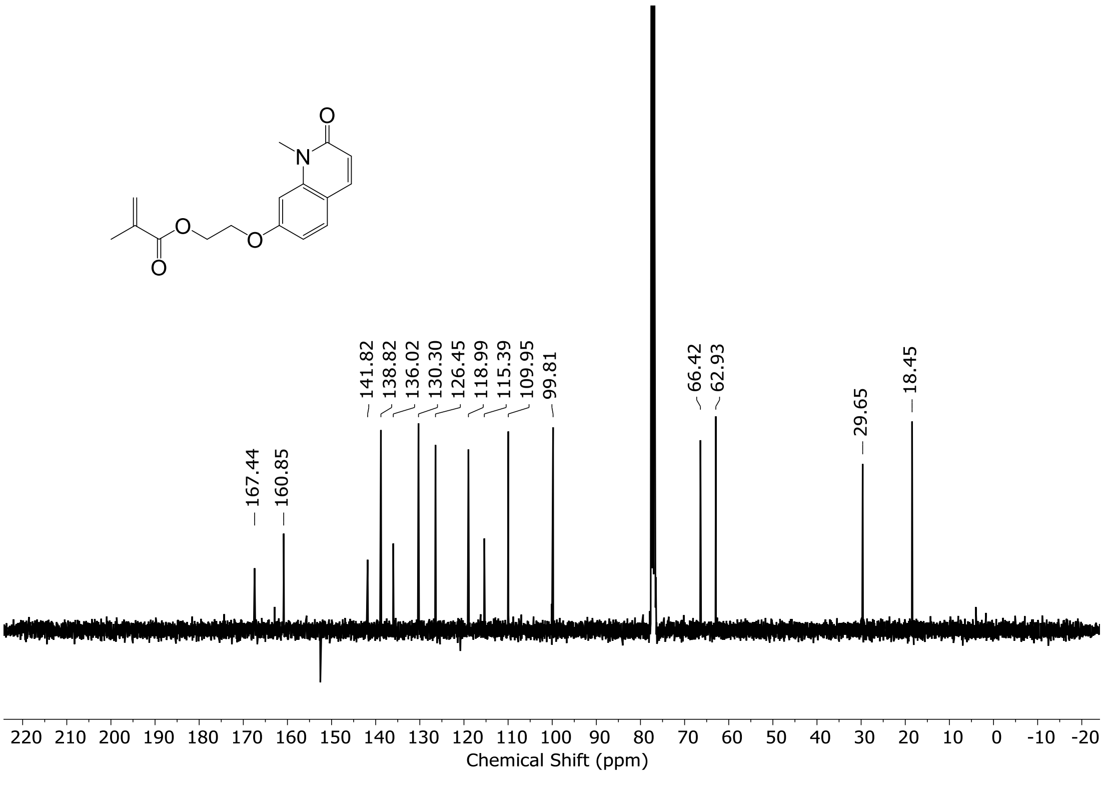

^{1}H-NMR spectrum of **PEHMA** in $CDCl_3$.

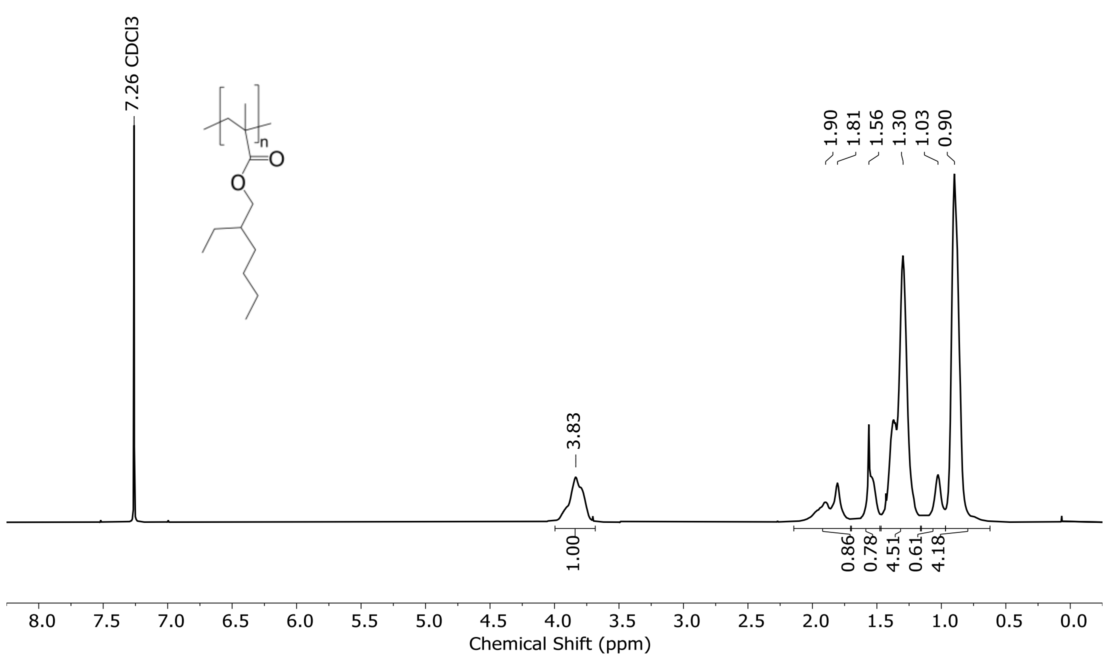

^{1}H-NMR spectrum of **PQM** in $CDCl_3$.

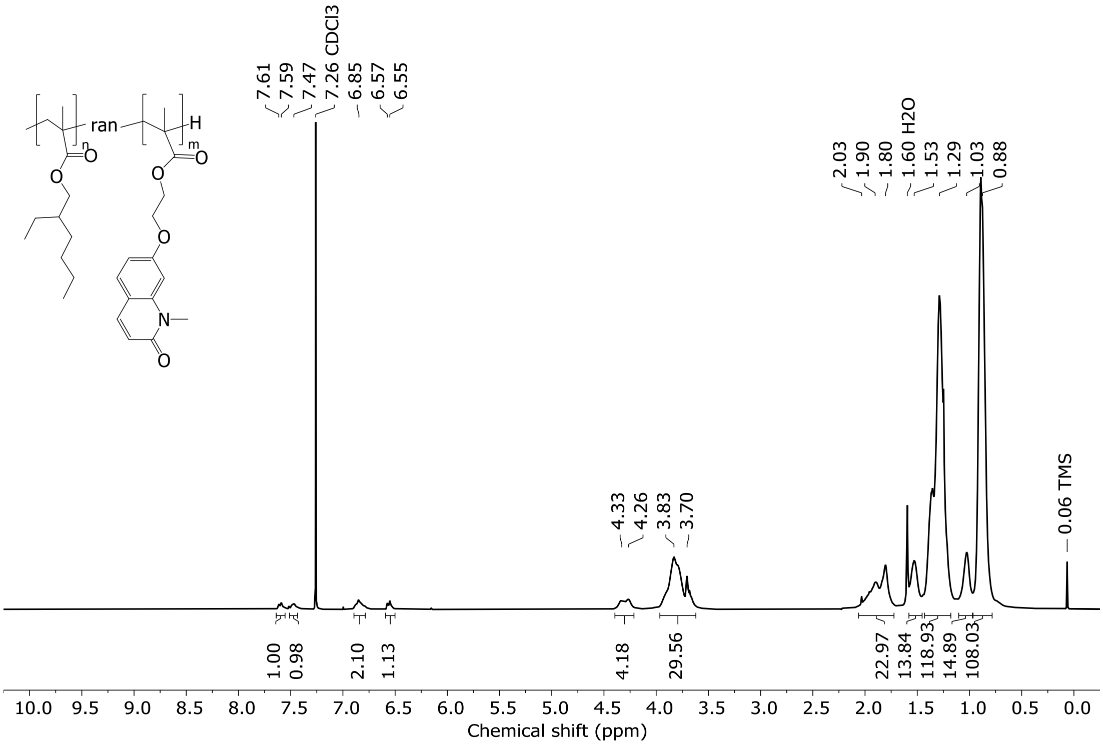

To determine the molar ratio of QMMA to EHMA repeating units in the copolymer PQM, quantitative analysis of the ^{1}H NMR spectrum was performed. The integral of the signal of the ^{1}H NMR spectrum ($I(\delta)$) of PQM in the range of 0.25 to 4.75 ppm is caused by 12 protons of the QMMA repeating unit and 22 protons of the EHMA repeating unit. It can be expressed as:

$$A_1 = \int_{0.25\text{ ppm}}^{4.75\text{ ppm}} I(\delta)\, d\delta = (n_{\text{QMMA}}12 + n_{\text{EHMA}}22)/\text{n}$$

where n_{QMMA} is the number of QMMA repeating units and n_{EHMA} is the number of EHMA repeating units in a polymer consisting of n total repeating units. The sum of the integrals in the range of 6.45 to 7.01 ppm and 7.35 to 7.68 ppm is attributed to the five aromatic protons of the QMMA unit and can therefore be described by the following equation:

$$A_1 = \int_{6.45\text{ ppm}}^{7.01\text{ ppm}} I(\delta)\, d\delta + \int_{7.35\text{ ppm}}^{7.68\text{ ppm}} I(\delta)\, d\delta = n_{\text{QMMA}}5$$

Using both equations, the ratios n_{QMMA} / n and n_{EHMA} can be determined. This allows the calculation of the molar ratio between the two repeating units (n_{EHMA} / n_{QMMA}), which is found to be 11.8.

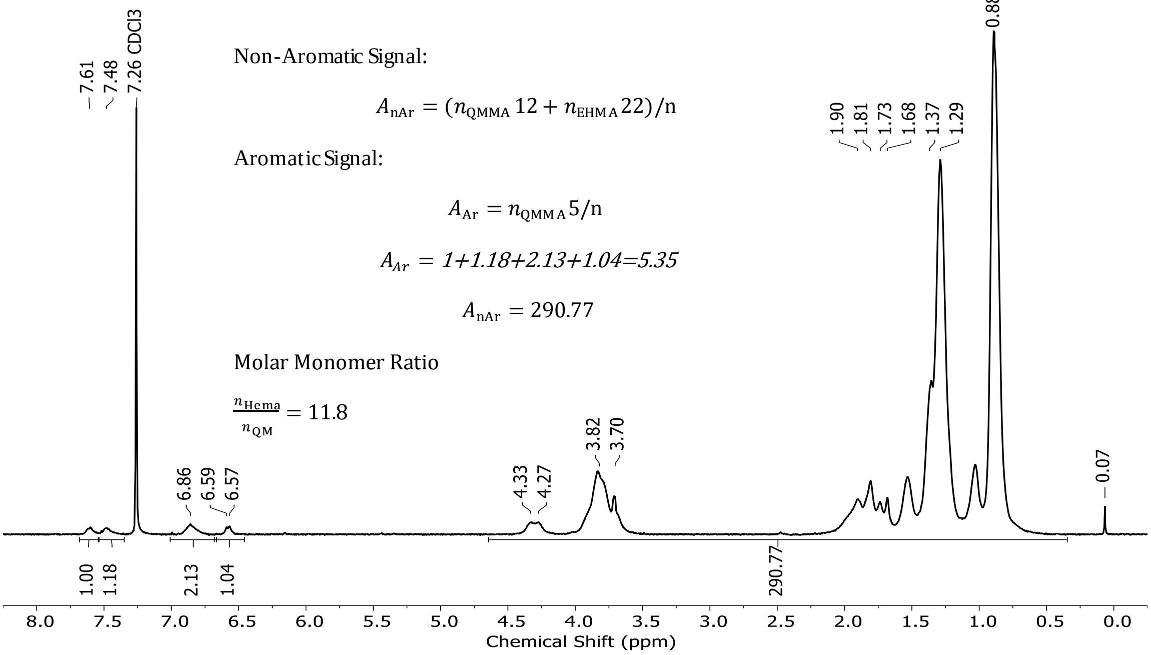

References

[1] H. Hertz, *J reine und angewandte Mathematik* **1881**, *92*, 156.

[2] P. H. Mott, J. R. Dorgan, C. M. Roland, *J Sound Vib* **2008**, *312*, 572–575.

[3] M. Krieg, G. Fläschner, D. Alsteens, B. M. Gaub, W. H. Roos, G. J. L. Wuite, H. E. Gaub, C. Gerber, Y. F. Dufrêne, D. J. Müller, *Nature Reviews Physics* **2019**, *1*, 41–57.

[4] M. Streicher, C. Stamp, M. D. Kluth, A. Ripp, C. Calvino, *Macromol Rapid Commun* **2024**, 2400474.
